# The impact of playlist characteristics on coherence in user-curated music playlists

**DOI:** 10.1140/epjds/s13688-025-00531-3

**Published:** 2025-03-19

**Authors:** Harald Schweiger, Emilia Parada-Cabaleiro, Markus Schedl

**Affiliations:** 1 Multimedia Mining and Search Group, Institute of Computational Perception, Johannes Kepler University Linz, Altenberger Straße 69, Linz, 4040 Upper Austria Austria; 2 Department of Music Pedagogy, Nuremberg University of Music, Veilhofstraße 34, Nuremberg, 90489 Bavaria Germany; 3 Human-centered AI Group, AI Lab, Linz Institute of Technology, Altenberger Straße 69, Linz, 4040 Upper Austria Austria

**Keywords:** Music playlists, Coherence, Correlation, Causality

## Abstract

Music playlist creation is a crucial, yet not fully explored task in music data mining and music information retrieval. Previous studies have largely focused on investigating diversity, popularity, and serendipity of tracks in human- or machine-generated playlists. However, the concept of playlist coherence – vaguely defined as smooth transitions between tracks – remains poorly understood and even lacks a standardized definition. In this paper, we provide a formal definition for measuring playlist coherence based on the sequential ordering of tracks, offering a more interpretable measurement compared to existing literature, and allowing for comparisons between playlists with different musical styles. The presented formal framework to measure coherence is applied to analyze a substantial dataset of user-generated playlists, examining how various playlist characteristics influence coherence. We identified four key attributes: playlist length, number of edits, track popularity, and collaborative playlist curation as potential influencing factors. Using correlation and causal inference models, the impact of these attributes on coherence across ten auditory and one metadata feature are assessed. Our findings indicate that these attributes influence playlist coherence to varying extents. Longer playlists tend to exhibit higher coherence, whereas playlists dominated by popular tracks or those extensively modified by users show reduced coherence. In contrast, collaborative playlist curation yielded mixed results. The insights from this study have practical implications for enhancing recommendation tasks, such as automatic playlist generation and continuation, beyond traditional accuracy metrics. As a demonstration of these findings, we propose a simple greedy algorithm that reorganizes playlists to align coherence with observed trends.

**Supplementary Information:**

The online version contains supplementary material available at 10.1140/epjds/s13688-025-00531-3.

## Introduction

Over the past few decades, music consumption has undergone continuous transformation. Formats like shellac records, tapes, and CDs have empowered individuals to listen to music at their convenience. The widespread availability of physical music media enabled music enthusiasts to curate and share collections of tracks with like-minded individuals, often in the form of mixtapes.

With the rise of online music streaming services, a new facet to the creation of music collections emerged, marking a shift from physical music collections to the realm of digital playlists. Playlists became independent of the constraints imposed by physical media, enabling users to craft track collections for purely personal reasons, sometimes only understandable for the creators themselves [1–3].

One subject of interest in music information retrieval (MIR) is the importance of track order in the process of curating playlists [4–6]. Specifically, the question arises as to what extent tracks should deviate within a playlist from the previous one(s) in terms of their musical and metadata properties to enhance the curator’s or listener’s experience. The paper at hand focuses on how playlist curators select and reorder tracks to achieve either a smooth or an exciting experience (e.g., to sudden changes in music style), a concept referred to as *coherence*.

A closely related area of investigation is playlist diversity, which is generally more well-defined and well-researched [7–13]. Playlist diversity describes the extent to which tracks differ within playlists without considering the sequential order. In contrast, coherence focuses on the smoothness or logical flow between tracks, taking into account the listening experience of users when they listen to the playlist in sequence.

For example, a playlist used for exercising might start with slow tracks in terms of beats per minute (BPM) to give the listener time for the warm-up. Subsequent tracks increase the tempo to fit the workout routine, and the final tracks of the playlist might slow down in terms of BPM to reflect the listener’s transition into the relaxation phase. Intuitively, the training playlist is considered coherent in terms of tempo, as there are no radical changes in BPM between subsequent tracks. At the same time, the playlist is also considered diverse in tempo, as it features tracks with varying BPM throughout the playlist.

In contrast, a playlist containing Dubstep music^1^ will likely contain tracks with a consistent tempo of around 140 BPM, which is typical for this genre. Thus, the Dubstep playlist is consistent (the opposite of diverse) as every track is similar in tempo. One key difference between a consistent and coherent playlist is that the former can be listened to in random order without largely affecting the listener’s experience. The coherent playlist might be diverse in the selected property under observation, but it can still deliver a smooth listening experience (similarly to a consistent playlist) if the tracks are arranged accordingly. Notably, since the arrangement of tracks is a criterion for a coherent listening experience, coherence is linked to the question of importance of track order within playlists.

While consistency and coherence are terms often used interchangeably in related research, in our work, coherence reflects the experienced smoothness between subsequent tracks in relation to the overall diversity of the playlist. More generally, we define coherence for sequential data as follows:

### Definition 1

(Coherence)

Disparity between deviation in selected properties of items in the entire sequence and deviation of these properties among items in local proximity.

Applied to playlists, the *entire sequence* refers to all tracks throughout the playlist, while *local proximity* pertains to subsequent tracks within a small, arbitrary window. According to this definition, coherence can also be interpreted as the deviation between the overall diversity of the playlist and the diversity on subsequent tracks. The formal relationships between diversity, coherence, variance, pairwise distances, and other related concepts are further elaborated in Sect. 4.

In music recommender systems (MRS), recommending suitable tracks to either generate a playlist, i.e., automatic playlist generation (APG), or extend an existing one, i.e., automatic playlist continuation (APC), are major research areas in MIR. However, simply recommending tracks that match the current context [14] or intent [15] of a playlist is not enough to provide a satisfying user experience. Recommendations that are too similar can be perceived as boring [16], which has been shown to negatively impact user conversion and retention on online streaming platforms [17].

While playlist diversity [11–13] and consistency/homogeneity [18–20] have been extensively studied in MIR, coherence, as a sequential and order-dependent property, remains underrepresented. User studies [2, 4] indicate that coherence is perceived as an important quality criterion. Coherence has also been identified as a challenging area that requires further investigation to ensure the quality of music recommender systems [21]. To demonstrate how a deeper understanding of coherence can enhance recommendations, we provide the following three examples:

### Example 1

In APG, playlists are created based on a desired number of tracks and additional constraints, such as seed tracks. If the recommendation system can determine the appropriate degree of coherence based on input parameters (in this case the playlist length) it can optimize its recommendations in a secondary stage by reordering or replacing tracks to achieve the targeted coherence degree.

### Example 2

In APC, the goal is to extend an existing playlist by recommending tracks that transition well from the current final track. In these cases, additional information, e.g., the popularity of tracks, can be retrieved to recommend suitable candidates [22]. Understanding how popularity and similar characteristics influence coherence can be used to rerank the track recommendation list, ensuring that the playlist remains engaging in the long term, particularly when playlist continuation is used repeatedly. Deriving coherence from factors like popularity is especially valuable when a playlist contains too few tracks to directly infer the desired coherence degree from the track sequence itself.

### Example 3

Understanding coherence and its relationship with other playlist characteristics can be applied to tasks beyond track recommendations. For instance, users often invest time reordering and modifying their playlists [23]. This process, however, can be fully automated by rearranging the playlist or simplified by suggesting additional tracks at specific points within the playlist to achieve a desired coherence score. To implement this effectively, it is essential to first understand how the coherence of a playlist evolves as users make modifications.

To demonstrate the applicability of these ideas into practical perspective, a simple greedy algorithm is proposed which rearranges the tracks of an existing playlist to align them with the coherence trends identified in this study. However, the primary contribution of this work lies in the definition and measurement of coherence, as well as providing a detailed analysis of the playlist properties that influence coherence. The playlist attributes to be analyzed are the *playlist length*, describing the number of tracks within the playlist, *track popularity*, defined as the average popularity of tracks within playlists, and *number of edits*, measuring how often a playlist has been modified over its lifetime (cf. Examples 1, 2, and 3 for the practical applications, respectively). These attributes have already been found to influence playlist diversity and play a key role in the general curation process of online users (cf. Sect. 3.2), but to the best of our knowledge, they have never been studied in the context of coherence.

Additionally, we aim to contribute to the growing trend of group-curated playlists by analyzing whether *collaborative playlists* differ from playlists with only one curator in terms of coherence. Although the ability to curate playlists collaboratively has been available on music streaming platforms for over a decade, this feature has received little attention. However, the demand for creating playlists as an online group activity has gained momentum since the onset of the COVID-19 pandemic, sparking increased research interest [24, 25].

In summary, this work assesses which properties of user-generated music playlists influence the coherence of their constituting tracks by addressing the following research questions (RQs):

### RQ 1

To what extent does playlist length influence playlist coherence?

### RQ 2

How does the popularity of tracks within a playlist impact coherence?

### RQ 3

Do dynamic playlists, which are continuously updated and modified, affect coherence differently compared to static ones?

### RQ 4

Do collaborative playlists, curated by more than one user, differ in terms of coherence?

The research questions are investigated by applying *correlation analysis* and *causal inference* to measure coherence differences across ten individual *audio features* and one *metadata feature* of tracks for over 650,000 playlists. This approach identifies general coherence trends and assesses the impact of individual playlist attributes while controlling for confounding factors. Addressing these questions should contribute to improving music recommendation systems, understanding user curation processes, and establishing a solid framework for future coherence analysis research.

The remainder of the paper is structured as follows: Sect. 2 presents previous research on track order, coherence, and other related topics. Section 3 introduces the analyzed dataset along with the evaluated playlist attributes as well as the features used to measure coherence. Section 4 establishes a formal definition of coherence as a derivation of variance. Section 5 describes the experimental setup, while Sect. 6 presents and discusses the results. Section 7 applies these findings in a proposed greedy algorithm for rearranging tracks in an existing playlist, illustrated with a specific example. Finally, Sect. 8 summarizes the findings and highlights possible future work.

## Related work

Playlist coherence is a topic that reappears in MIR-related research under different names, e.g., track order, track sequencing, consistency or smoothness of track transition. As there is no uniform definition of coherence, its understanding, approach, and measurement can vary significantly across studies. For instance, [7] uses the term coherence to refer to the concept of consistency/homogeneity, while [18] uses the term consistency in a way that aligns more closely with our definition of coherence.

Generally, four types of approaches can be identified: 1) User studies, which quantify the importance of coherence by interviewing participants, evaluating questionnaire responses, or analyzing blog posts. 2) Offline experiments, evaluating the performance differences between sequential and order-agnostic algorithms. 3) Proposals for automatic playlist generation (APG) and continuation (APC) that use sequential or order-aware models, thereby indirectly claiming that track order (and coherence) are important criteria. 4) Research analyzing datasets for evidence of coherence.

### User studies

A major contribution to understanding the curation process is the user study by [2], which provides several findings related to coherence: certain tracks belong together in some context and should be played in sequence; consecutive tracks should have complementary styles (though this rule can be broken to play with the listener’s expectations); and reordering, deleting, as well as replacing tracks, occurs after establishing the tracks within the playlist. This research also shows that it is important that not too many tracks of the same kind/artist follow in sequence, and that both, the first and final tracks, serve special purposes. Finally, the research also highlights that users might listen to the same playlist in random order (shuffle mode) to keep the playlist “fresh” or achieve serendipity. The advantages and disadvantages of a shuffled listening experience are also covered by [26, 27]. Differently, in other post-experimental surveys, participants indicated that track order does not necessarily influence the perceived quality of playlists. Similarly, in [6], half of the participants agreed that track order does not matter to them if they like the tracks, and in [9], participants did not even expect recommendations to be in a specific order. In [28], two-thirds of participants mentioned that track order is important, especially for creating sequences that blend well together. Similarly, in user studies by [4, 5, 23], participants also reported that track order and transition between tracks are important; still, this aspect ranked lower compared to other criteria.

### Offline experiments on APG & APC

Adopting offline experiments also provides insights into track order. For instance, [22] trained a recurrent neural network (RNN) for sequential recommendation on original and shuffled data sets and found no performance difference, questioning the importance of track order. An earlier experiment by [29] concluded that their Markov chain approach, which produces coherent playlists using audio and social-tag similarities, performed worse than order-agnostic approaches. However, in a subsequent experiment using hypergraph random walks, the same authors found that including track transition information improved performance [30]. Similarly, [31] found track transitions important as well, with the additional finding that reversing the track order achieves comparable results.

### Coherence-related APG & APC

Other research assumes that smooth transitions or track order are valuable quality criteria. For example, [32, 33] propose procedures to fill a sequence with tracks between a start and end track to minimize track-to-track transition differences. [34, 35] aims to reorder tracks to achieve smooth transitions. [8] proposes a hybrid recommendation system that balances coherence and diversity based on given tracks and similar playlists. [18] proposes a consistency-aware gating network that evaluates if the last track in the playlist deviates from previous ones, and adjusts recommendations accordingly.

### Dataset analysis

In the analysis of existing playlist datasets, [13] showed that consecutive tracks are more similar in the first half of playlists compared to the second half in terms of artist, genre, and other features. [36] observed that long playlists feature repeated patterns of the same artists and albums. An analysis by [37] demonstrated that tracks closely positioned in user-generated playlists are more similar than those further apart according to the investigated audio features (i.e., *loudness*, *energy*, *danceability*, *acousticness*, *valence*, *speechiness*, *tempo*, *tonality*, *liveness*, and *loudness*) and metadata (i.e., *genre*, *artist*, *popularity*).

### Research contribution

The importance of coherence is evaluated with varying relevance in existing literature. According to previous work, individual user preference and listening intent are contributing factors to whether and to what extent coherence plays a role [2, 5, 7]. However, this high-level information is often not obtainable for playlists, questioning how these factors translate to measurable playlist characteristics.

In our work, we aim to bridge this semantic gap by identifying playlist attributes that are decisive for creating coherent playlists. As these attributes describe measurable playlist characteristics, findings can be directly translated to practical applications (cf. Example 1, 2, and 3). To the best of our knowledge, no research has yet investigated how playlist coherence is causally affected by certain playlist characteristics, making this work a pioneering effort in coherence analysis.

Furthermore, we propose a framework capable of measuring coherence for any playlist, as long as track features are available to which a distance metric can be applied. This framework is independent of the type of music contained within the playlist. While existing literature measures playlist coherence by averaging the distances between subsequent tracks [8, 13], our approach introduces an additional normalization and transformation step that estimates coherence based on track arrangement. These steps standardize our measurement on a fixed scale from −1 to 1, offering the advantage that results from different distance metrics become directly comparable, e.g., coherence scores for tempo and loudness can be compared, even though these metrics capture different musical characteristics.

## Materials and features

This section provides details on the dataset used for coherence analysis (Sect. 3.1), the metadata of playlists that may influence coherence (Sect. 3.2), and the track features used to measure playlist coherence. Coherence calculations are conducted using nine continuous and a combination of two categorical audio features (Sect. 3.3). Additionally, coherence is measured using artist information to offer a metadata centered perspective (Sect. 4.7).

### Million playlist dataset

**Figure 1:**
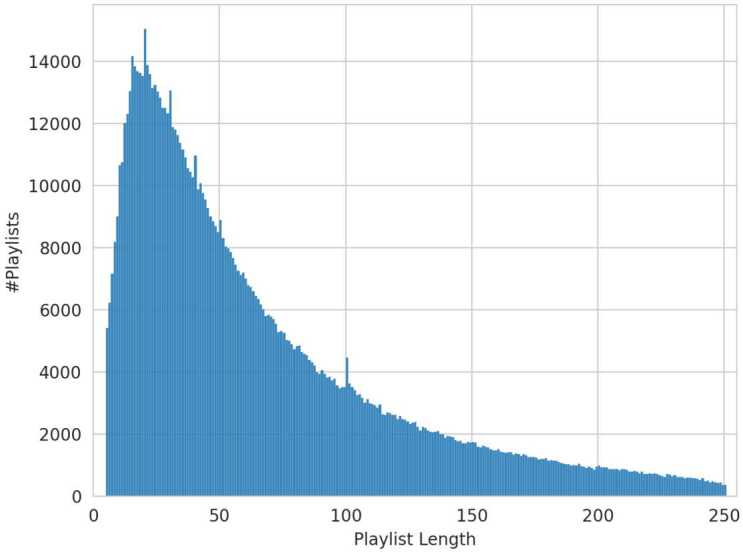
Distribution of Track Counts in Playlists

To address our research questions, we utilized the Million Playlist Dataset (MPD), made available by Spotify for the ACM Recommender Systems Challenge in 2018 [38]. This dataset comprises one million user-generated playlists created by individuals in the United States. The playlists vary in length, ranging from 5 to 250 tracks, with an average track count of 66 per playlist. The distribution adheres to a smooth yet skewed pattern, as illustrated in Fig. 1, with notable peaks of length 15, 20, 30, 40, 50, and 100. In total, there are 2,262,292 distinct tracks.

The dataset exclusively includes playlists that were shared publicly from January 2010 to November 2017. Furthermore, each playlist in the dataset contains a minimum of three unique artists and two unique albums.

We selected this dataset because it is the largest publicly available collection of human-curated playlists for research purposes. The creation process of playlists is multifaceted, influenced by factors such as the intended purpose and the music taste of the creator [1–3]. Given this background and its size, the MPD is a suitable choice for extracting and analyzing subtle trends, such as coherence, in the human curation process.

### Playlist attributes

The MPD provides various information for each playlist, including the playlist title, the playlist length, the time of the last modification, the number of playlist followers, as well as the IDs, names and albums of tracks contained within each playlist along with other metadata. This section focuses on playlist attributes that may influence playlist coherence. These attributes have been identified based on previous research, which observed differences in user curation behavior, particularly concerning diversity (a concept closely linked to coherence).

The *playlist length* reflects the number of tracks within a playlist. This is relevant since previous research has shown that longer playlists tend to be more diverse, e.g., to keep the listener engaged [12].

The *number of edits* attribute^2^ indicates the number of editing sessions in which the curator made modifications to the playlist, i.e., adding new tracks, deleting old ones, or reordering parts of the playlist. During a session, the user can make multiple changes to the playlist without increasing the edit score. An editing session ends if the user does not make any modifications for at least two hours. Notably, this score is not affected by any listening events of the curator.

**Figure 2:**
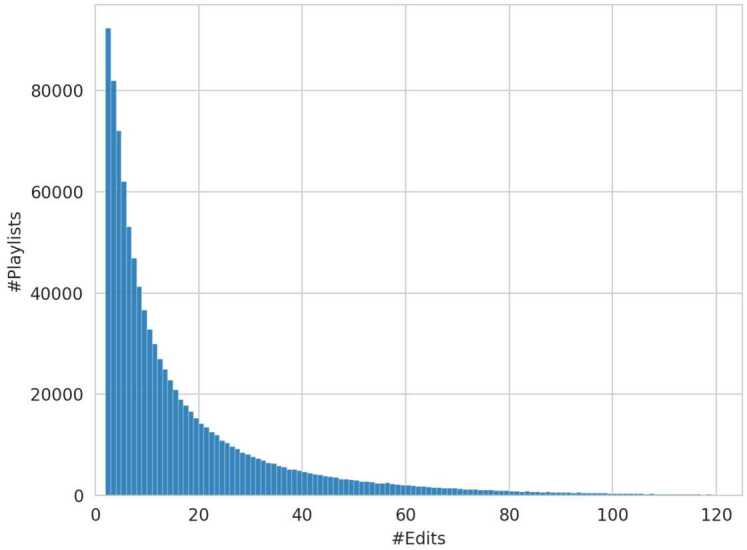
Number of Edits Distribution. The distribution shows the number of editing sessions performed by curators across playlists. Note that #Edits extend up to 201, but higher values are omitted for visual clarity

Research has shown that playlists differ in structure and purpose, depending on the modifications applied [3]. A playlist can become static, i.e., no further changes are made if the curator feels the playlist is complete or if it was created for a specific event. In these cases, the curator might still listen to the playlist but will not make further modifications. This typically results in fewer editing sessions compared to dynamic playlists. Dynamic playlists are continuously maintained and updated to stay fresh and align with the listener’s current music taste. In our experiments, dynamic playlists are further subdivided into two categories, *dynamic* and *highly dynamic* playlist, to access this attribute on a more granular level. The separation between static, dynamic and highly dynamic playlists is based on the number of editing sessions as detailed in Sect. 5.1. Figure 2 shows the distribution of all playlists in the MPD according to the number of editing sessions, revealing a pronounced long-tail distribution. Notably, the MPD primarily contains playlists with two or more editing sessions due to quality constraints imposed during the dataset’s creation.

Previous research has shown that some users specifically align their playlists according to the popularity of tracks [4, 23], and that incorporating *track popularity* into recommendations can improve performance [22, 29, 31]. A related topic, *mainstreamness*, has been identified as being in tension with diversity, as factors such as artist familiarity, genre affiliation, and similarity in audio features contribute to tracks being placed in the top charts [39]. Based on these findings, we hypothesize that track popularity may also be in tension with playlist coherence.

**Figure 3:**
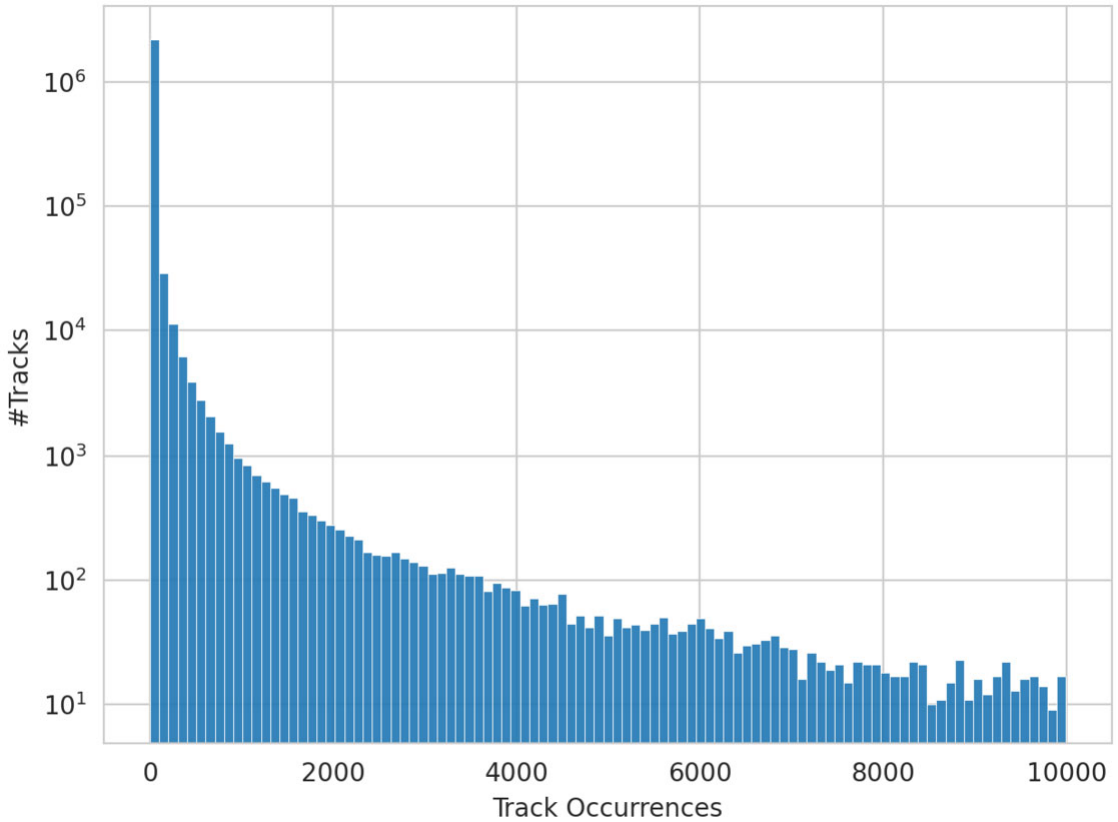
Track Count Distribution. The frequency with which individual tracks appear throughout the dataset. The most frequent track appears 46,574 times. Additionally, the y-axis is logarithmically scaled due to the pronounced long tail

To translate track popularity^3^ into a playlist property for subsequent coherence analysis, the following steps are performed. The number of occurrences of individual tracks throughout the dataset is counted. In line with common patterns observed in popularity data, the resulting distribution of track occurrences exhibits a long-tail distribution with 47.45% of all tracks appearing only once across the MPD, as shown by the log-scaled y-axis in Fig. 3.

**Figure 4:**
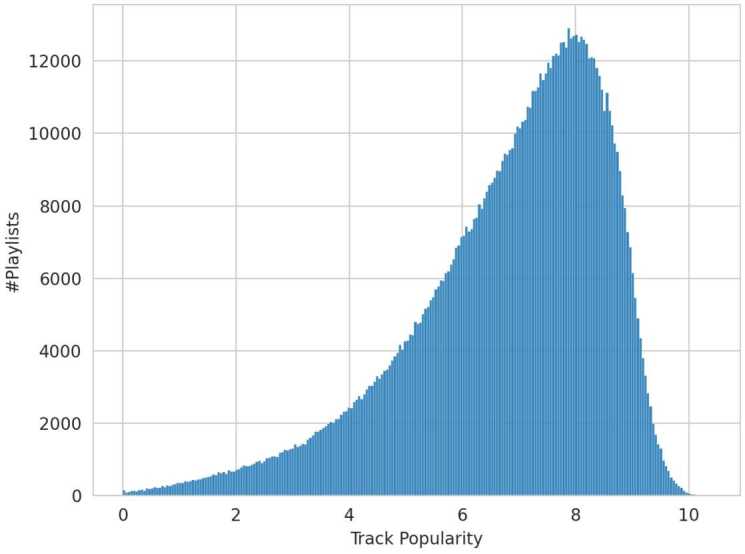
Track Popularity Distribution. Distribution of the playlist attribute “track popularity”, calculated by scaling and averaging the popularity of tracks within each playlist

Consequently, the track counts are scaled using the natural logarithm to mitigate the disproportionate impact of highly popular tracks and to ensure a more balanced distribution. Afterward, the scaled track counts are averaged and assigned as the track popularity attribute for each playlist. From this point forward, track popularity will refer to the scaled and averaged value of this playlist attribute. The resulting distribution of the track popularity attribute, calculated individually for each playlist, is depicted in Fig. 4.

The MPD provides information on whether a playlist is marked as collaborative. A playlist becomes a *collaborative playlist* (CP) when any user, in addition to the creator, is allowed to modify the playlist, i.e., after another user has gone through the invitation and joining process for that playlist. When a playlist is not a CP, we will refer to it as a *personal playlist* (PP).^4^ CPs can lead to playlists containing more diverse music, as the musical tastes of multiple users feed into one playlist [24, 40]. However, the track selection process can also become more rigid if curators choose tracks that are guaranteed to please all curators’ musical tastes [41].

Although the proportion of collaborative versus personal playlists on music streaming platforms is unknown, previous research [25, 40] revealed that 58% and 68% of US Spotify users, respectively, across two user studies, have created or contributed to CPs at least once in their lifetime. This high proportion of users engaging with CPs highlights the importance of analyzing this attribute in the context of this work.

Unfortunately, measuring the ratio of users contributing to CPs and PPs is not feasible in the Million Playlist Dataset (MPD) due to the lack of user information and the removal of references to original playlist IDs on Spotify’s platform. However, it is possible to calculate the proportion of CPs and PPs based on the total number of playlists, rather than the number of users involved. In the MPD, 2.26% of all playlists, totaling 22,569, are marked as collaborative. This percentage is notably lower than the user ratio provided by [25, 40] might suggest. However, the ratio of users who have contributed to CPs cannot be directly mapped to the proportion of CPs in the dataset, as users can create multiple PPs, while a single CP can involve contributions from several users. The discrepancy may also partially stem from higher engagement levels among survey respondents and the time gap between the survey and the MPD’s data collection, as CPs appear to be a more recent trend.

### Audio features

To measure coherence, three types of features are collected for each track: continuous and discrete audio features, representing various musical concepts as well as metadata on the artists involved in the recording. A summary of all features used for coherence measurement is provided in Table 1.

**Table 1:** Overview of Features with Their Respective Data Type

Features	Data type	Scale	Transformation	Distance
danceability, energy, valence, speechiness, instrumentalness, liveness, acousticness	continuous	[0,1]	None	Euclidian
loudness	continuous	[−60,5]	min-max-scaling	Euclidian
tempo	continuous	0∧[30,250]	min-max-scaling	Euclidian
mode	nominal	{0,1}	* combined into joint 2D space	tonality
key	nominal	{0,1,…,11}		
artist	set of ids		list of sparse vectors	averaged cosine

The scale informs about the initial numeric range retrieved from the Spotify API. Since some features are normalized, this is indicated in column transformation. Furthermore, the distance functions used for coherence calculation are summarized in the distance column (cf. Sect. 4 for more details). (*) *key* and *mode* are combined during coherence calculation and act as one feature.

Since playlist coherence is based on the observed changes across a track sequence, having high coverage of tracks in terms of feature information is mandatory. Without this coverage, only fragmented subsequences of the playlist could be analyzed. Therefore, it is advantageous to retrieve feature information from the same origin as the dataset. Originally developed under the name Echo Nest, Spotify provides 11 distinct high-level audio features [42] that have been widely utilized in numerous studies [43–48]. The exact process of how Spotify extracts these 11 features is proprietary and not publicly disclosed. However, according to the works and blog posts of the founders Tristan Jehan [49] and Brian Whitman [50], the features are derived through various signal processing techniques on auditory data, which are combined to form high-level features. It is also mentioned that machine learning algorithms are employed and fed with user-generated track descriptions [51, 52]. In total, there are 11 audio features, with some describing well-defined musicological concepts and others referring to more intuitive but abstract concepts.

The feature *danceability* describes the suitability of a track for dancing, combining aspects such as tempo, rhythm, beat strength, and beat regularity [53].

*Energy* integrates elements like loudness, timbre, onset rate, and general entropy of a track to capture its perceptual intensity and activity. Tracks that are perceived as fast, noisy, and loud score close to one, while tracks with calm music (often for solo instruments, as common in the classical repertoire) score closer to zero. This feature is related to the arousal dimension of Russell’s circumplex model of affect [54].

The second dimension of Russell’s circumplex model of affect [54] is *valence*, which is provided by Spotify under the same name. Valence describes the general positivity of a track, with values close to one for happy, cheerful, and euphoric tracks, while low scores are given to tracks perceived as sad, depressed, or angry.

*Speechiness* measures the amount of spoken words in a track. Talk shows, audiobooks, and poetry generally score above 0.66. Tracks with both spoken words and music scores between 0.33 and 0.66, while tracks primarily consisting in instrumental music score closer to zero. Notably, pure vocals do not significantly raise the score.

The *instrumentalness* feature indicates whether a track contains vocals, excluding non-lexical vocables (e.g., “Ohh” and “Ahh” sounds). It provides a confidence score for the absence of vocals rather than their relative usage throughout the track, leading to values close to either zero or one.

*Liveness* measures the probability that a track was recorded in front of an audience.

*Acousticness* assesses the presence of natural sounds versus electrical instruments. Tracks with acoustic instruments and unprocessed human voices score closer to one, while tracks with electric guitars, synthesizers, auto-tuned vocals, and drum machines score lower.

The remaining features are more *musicologically* well-defined concepts:

*Tempo* measures the BPM, ranging from 30 to 250. Notably, 2703 tracks have a BPM of 0, primarily tracks without music, such as commentary. However, there are also tracks that are assigned with a BPM value, even if they do not contain music such as audiobooks. Since tempo does not fall into the value range between zero and one, min-max scaling is applied before the coherence calculation is performed.

*Loudness*, from a musicological point of view related to the concept of *dynamics*, quantifies how loud or quiet a track sounds to the human ear, measured by the average strength of the track’s amplitudes, with a range from −60 dB to 0 dB. While 0 dB typically represents the maximum loudness without distortion, a few tracks have a loudness value close to 5 dB. Similarly to *tempo*, the values of the *loudness* features are also transformed using min-max scaling.

Spotify’s API also provides categorical audio features like *mode* (major and minor) and *key* (pitch class). Since these values are categorical with nominal scale and are linked to each other from a musicological perspective, i.e., defining the *tonality*, they are used in a joint representation for measuring coherence as described in Sect. 4.6.

**Figure 5:**
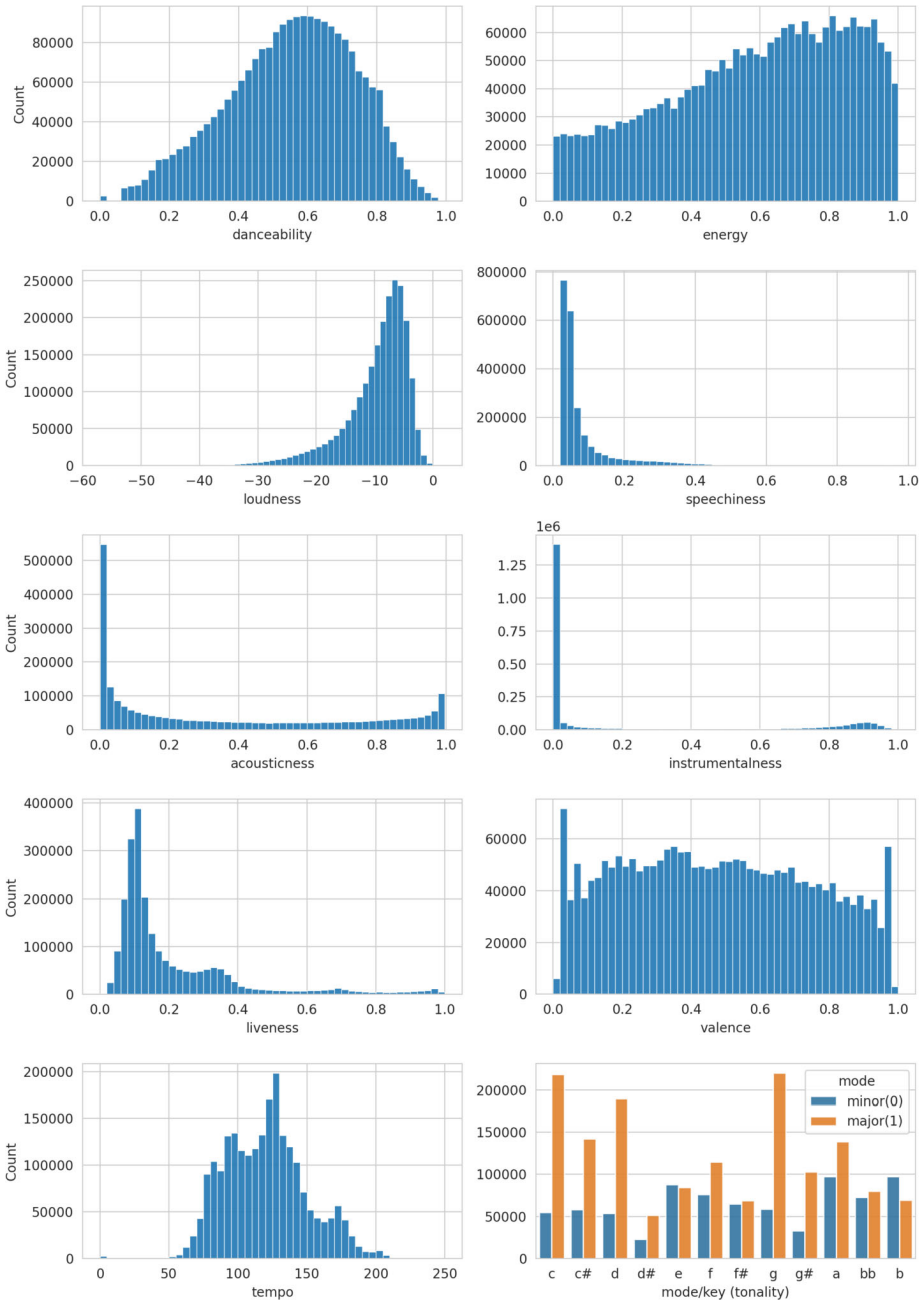
Audio Feature Distribution. Distribution of Spotify’s audio features measured across all individual tracks of the MPD

The distribution of the audio features is shown in Fig. 5. As visible, the spread of the values varies depending on the measured audio property, with features measuring confidence values, i.e., *speechiness*, *instrumentalness*, *liveness*, and *acousticness*, being less uniformly distributed. This is a relevant observation for coherence analysis, as the feature distribution can affect the shape of the respective coherence distribution, cf. Sect. 4.5.

### Artist feature

In addition to audio features, coherence is also measured from a metadata perspective using artist information. Previous work has shown that the artist of a track is an important factor during the curation process of playlists [4, 23, 36]. For instance, [2] noted that tracks to be added should fit or avoid certain artists. Some playlists are even centered around a specific artist, who might also be featured on the cover. In the same study, a guideline of not including tracks from the same artist more than once has been mentioned. Other experimental studies have also shown improvements in track recommendations when artist information is provided [20, 36].

To analyze whether certain playlist attributes affect playlist coherence in terms of the artist selection and arrangement, differences in artist similarity between subsequent tracks are measured. In this context, the artist of a track refers to the main performer, e.g., the band. The dataset provides the main artists for each track. However, songs are often created in collaboration with other artists. Featured guest artists, often listed after “feat.” or “ft.” within the track title, perform important parts of a track, which would not be reflected using solely the provided data.

To include these additional artists, we issued queries for all tracks within the MPD to the Spotify API endpoint [55]. This expanded the initial set of 295,860 unique artists to a more comprehensive 411,080. The purpose of including all performers within a playlist is to capture also smooth transitions, such as when a song features a guest artist who becomes the main artist in the next track of the playlist.

Generally, the provided and queried artist information is used to establish a distance metric to measure the artist’s difference between tracks. This distance measurement is applied as described in Sect. 4.7.

## Methodology

For measuring the coherence within playlists, we took a similar approach to that presented in [37], which utilizes the derivations of deformation formulas on variance by [56]. In Sect. 4.1, the variance formula, being one part of the coherence formula, is described along with derivations that replace the need of calculating the mean using pairwise distances. This derivation allows for the creation of an order-dependent measurement of variance, denoted as sequential variance (cf. Sect. 4.2). Section 4.3 presents the final coherence formula and contextualizes its numeric range for scalar values (cf. Sect. 4.4). Section 4.5 analyzes distributions resulting from applying the coherence formula to MPD and derives key implications for the experimental setup. Finally, Sects. 4.6 and 4.7 describe the procedures for deriving and measuring coherence in terms of tonality (a combination of the audio features *mode* and *key*) and the metadata artist features, respectively.

### Variance

Let *X* be a random variable. The variance of *X* is defined as the expected squared deviation from the expected value of *X*:

1 $$ \text{Var}(X) = E[(X-E[X])^{2}] $$

Drawing a finite set of independent and equally weighted observations from *X*, denoted as $\mathbf{x} = \{x_{i} | i = 1, 2, \dots , n\}$ with *n* elements, the expected value of *X* can be estimated by calculating the sample mean *x̅*:

2 $$ \overline{x} = \frac{1}{n} \sum _{i=1}^{n} x_{i} $$

The population variance^5^$\sigma ^{2}$ is calculated by averaging the squared differences between each observation and the sample mean:

3 $$ \sigma ^{2} = \frac{1}{n} \sum _{i=1}^{n} (x_{i}-\overline{x})^{2} $$

Alternatively, the population variance can be reformulated without the sample mean [56] by calculating the average of all squared pairwise distances of *X* divided by two, as shown in Equation (4). This approach interprets variability as twice the expected deviation between two randomly drawn observations, measured by their squared pairwise distance:

4 $$ \sigma ^{2} = \frac{1}{n} \sum _{i=1}^{n} (x_{i}-\overline{x})^{2} = \frac{1}{2 n^{2}} \sum _{i=1}^{n} \sum _{j=1}^{n} (x_{i}-x_{j})^{2} $$

Since $(x_{i}-x_{j})^{2}$ is symmetric, this can be simplified to avoid redundant computations:

5 $$ \sigma ^{2} = \frac{1}{2 n^{2}} \sum _{i=1}^{n} \sum _{j=1}^{n} (x_{i}-x_{j})^{2} = \frac{1}{n^{2}} \sum _{i=1}^{n} \sum _{j=i+1}^{n} (x_{i}-x_{j})^{2} $$

While calculating the variance using pairwise distances is computationally more intensive, it also opens new ways of using other distance functions. For example, [57] utilized this technique to measure variability for categorical variables using the Kronecker delta as the distance function. Therefore, Equation (6) is a generalized variant to include other distance functions, denoted as *d*:

6 $$ \sigma ^{2} = \frac{1}{n^{2}} \sum _{i=1}^{n} \sum _{j=i+1}^{n} {d}(x_{i}, x_{j})^{2} $$

Notably, variability, unalikeability, and population diversity are equivalent concepts that can be found in disciplines such as sociology, economics, linguistics, and ecology [57].

For our purpose, when the population variance is calculated on scalar values, i.e., the continuous audio features, Equation (5) is used. For measuring variance for multidimensional features, i.e., for the tonality and artist feature, a different distance function is needed, cf. Sects. 4.6 and 4.7, respectively.

### Sequential variance

The variance discussed so far measures the general variability of an independent random variable. In contrast to the population variance $\sigma ^{2}$, the next variance formula, referred to as sequential variance, accounts for the order of the samples drawn. More specifically, the sequential variance $\overrightarrow{s}^{2}$ accounts for the variability observed by measuring the expected deviations from one sample to the next. To achieve this, the average distance^6^ between each item and its immediate successor is measured, rather than considering all possible pairwise combinations (cf. the population variance in Equation (4)):

7 $$ \overrightarrow{s}^{2} = \frac{1}{2 n} \sum _{i=1}^{n-1} (x_{i}-x_{i+1})^{2} $$

Additionally, the formula can be adapted to include other distance metrics as well:

8 $$ \overrightarrow{s}^{2} = \frac{1}{2 n} \sum _{i=1}^{n-1} d(x_{i}, x_{i+1})^{2} $$

One property of Equation (8) is that the average of $\overrightarrow{s}^{2}$ for a vast number of random permutations on the sequence **x** will approximate the population variance $\sigma ^{2}$.

### Coherence

Previous research has shown that playlists can differ in their diversity due to multifaceted factors, including individual preferences, genres or themes, and other contextual influences [2, 10–12]. As variance is a specific manifestation of diversity, the statement also holds for $\overrightarrow{s}^{2}$ and $\sigma ^{2}$. For example, metal music is mostly played on electrical instruments. As a result, the average variances for the *acousticness* feature are low, with $\overrightarrow{s}_{\text{metal}}^{2}=0.016$ and $\sigma _{\text{metal}}^{2}=0.017$ for metal playlists^7^ in the MPD. In contrast, playlists containing pop music alternate more often between acoustic and electrical instruments, leading to higher variances in *acousticness*, with $\overrightarrow{s}_{\text{pop}}^{2}=0.04$ and $\sigma _{\text{pop}}^{2}=0.043$.

Nevertheless, it would be misleading to assume that metal playlists are more coherent than pop playlists simply because $\overrightarrow{s}_{\text{metal}}^{2} < \overrightarrow{s}_{ \text{pop}}^{2}$. In fact, both genres reach nearly the same coherence score with $\text{coh}_{\text{metal}}=0.0594$ and $\text{coh}_{\text{pop}}=0.0598$, as calculated using by Equation (9). By measuring the sequential variance $\overrightarrow{s}^{2}$ and normalizing it by the overall diversity of the playlist $\sigma ^{2}$, enables the estimation of playlist coherence based on track arrangement, regardless of the specific tracks within the playlist. This approach facilitates comparisons of coherence across playlists with different musical themes.

Measuring coherence for scalar values using $\overrightarrow{s}^{2} / \sigma ^{2}$ produces results within the range of 0 to 2, with values close to 0 indicating highly coherent samples. However, since this scale may appear slightly counterintuitive, the formula is further transformed by changing the sign and shifting by 1. This adjustment aligns the scale with the following intuitive interpretation: Playlists that do not hold coherent properties, e.g., playlists where tracks are shuffled, average a value close to zero. Coherent playlists, i.e., playlists that vary sequentially less than the population variance would imply, have a value above zero. Playlists arranged to create mostly abrupt changes have a coherence value smaller than zero.

Given these properties, the coherence formula is defined by Equation (9):

9 $$ coh = 1 - \frac{\overrightarrow{s}^{2}}{\sigma ^{2}} = 1 - \frac{n}{2} \frac{\sum _{i=1}^{n-1} d(x_{i}, x_{i+1})^{2}}{\sum _{i=1}^{n} \sum _{j=i+1}^{n} d(x_{i}, x_{j})^{2}} $$

### Coherence bounds

Coherence values for scalar features are bounded within the range of −1 to 1. For sequences to approach these bounds, they must be sufficiently long, and the sequential variance $\overrightarrow{s}^{2}$ must be maximized or minimized, respectively.

An example of a sequence that maximizes the sequential variance is $[0, 1, 0, 1, \dots ]$. As demonstrated in Equation (10), for a sufficiently long sequence, the coherence value converges to −1. A formal proof for this example is provided in the supplementary materials under Lemma 1.

10 $$ coh([0, 1, 0, 1, \dots ])_{n \rightarrow \infty } = \lim _{n \rightarrow \infty } 1 - \frac{2(n-1)}{n} = 1 - 2 = -1 $$

Conversely, a sequence that maximizes coherence towards 1 is achieved by minimizing the distances between consecutive elements $x_{i}$ and $x_{i+1}$. For example, this condition is satisfied by a sequence that linearly interpolates between the first and last elements. The proof for this case is provided in the supplementary materials (cf. Lemma 2).

11 $$ coh([\frac{1}{n}, \frac{2}{n}, \dots , \frac{n-1}{n}, \frac{n}{n}])_{n \rightarrow \infty } = \lim _{n \rightarrow \infty } 1 - \frac{6}{n(n+1)} = 1 - 0 = 1 $$

### Coherence properties

In the following, two observations on distributions created by coherence measurements on playlists are highlighted, leading to two respective design decisions. Firstly, the Pearson correlation, which is mathematically equivalent to the point-biserial correlation, offers a more accurate method for quantifying the relationship between playlist attributes and coherence than alternative measures like Spearman’s Rho or Kendall’s Tau. Secondly, the average number of tracks influences the skewness of distributions and, more generally, the confidence intervals of the results. This necessitates the use of causal inference to account for confounding factors in playlist attributes that correlate with the playlist length.

#### Factors for skewness

Measuring coherence using Equation (9) across multiple playlists with randomly arranged tracks results, on average, in coherence values that center around zero for both the mean and the median. This property holds if at least one of the following two conditions is met:

1. The underlying feature, over which the coherence values are calculated, follows a normal distribution.
2. Coherence is calculated over a sufficiently long sequence.

**Figure 6:**
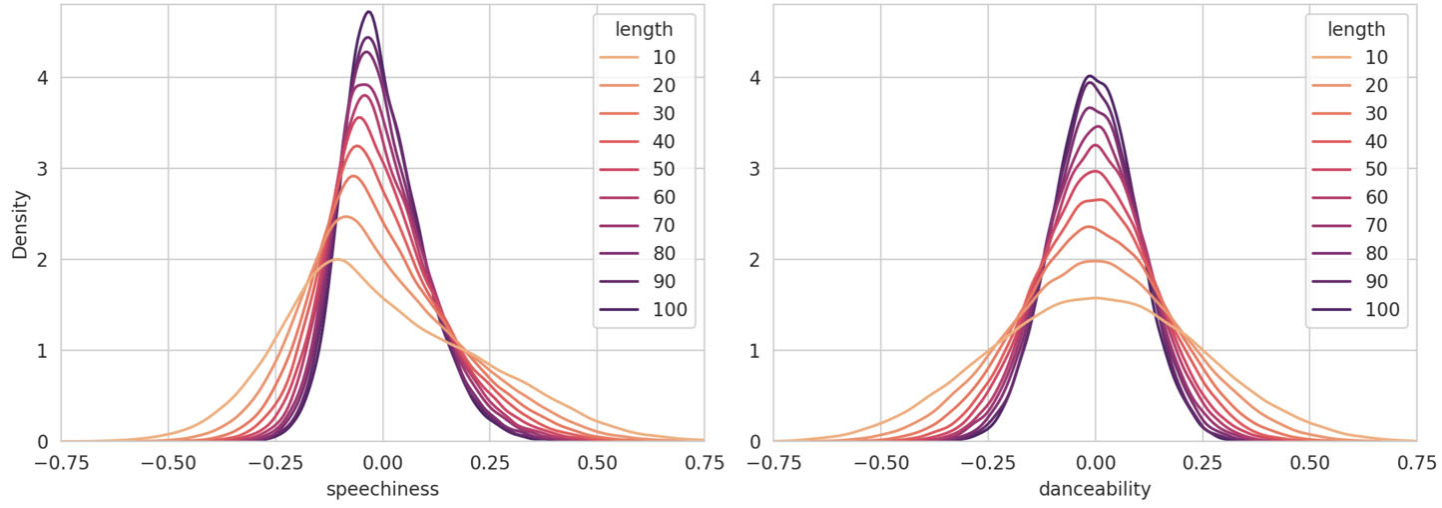
Stacked Density Plot of Coherences for the Audio Feature Speechiness and Danceability on Shuffled Playlists. The individual coherence distributions are grouped according to the playlist length, with a step size of 10. Each density plot is scaled so that the area under the curve sums to one

If neither of these conditions is fully satisfied, the coherence distribution becomes skewed, causing the median to deviate from zero. This effect is particularly pronounced when the underlying distribution deviates from a symmetric bell curve, such as in multimodal distributions with multiple peaks or in skewed distributions. Figure 6 illustrates this by calculating the coherence values for shuffled playlists for two audio features: *speechiness*, which follows more of a chi-square distribution, and *danceability*, which is closer to a normal distribution (cf. Fig. 5 for the feature distributions). The playlists have been sampled individually according to different playlist lengths. For short playlists, the *speechiness* coherence values are visibly asymmetric compared to those for *danceability*, resulting in more outliers on the positive side and a median that deviates from zero, cf. Fig. 6 left.

Despite these differences, the mean coherence remains centered around zero for both features, regardless of the underlying distribution. Moreover, as demonstrated in both plots in Fig. 6, coherence values can be estimated with greater confidence for longer playlists, which are less likely to exhibit high variability by chance. Additionally, the coherence of longer playlists tends to approximate a normal distribution, regardless of the underlying distribution of the feature.

#### Importance of Pearson & point-biserial correlation

**Figure 7:**
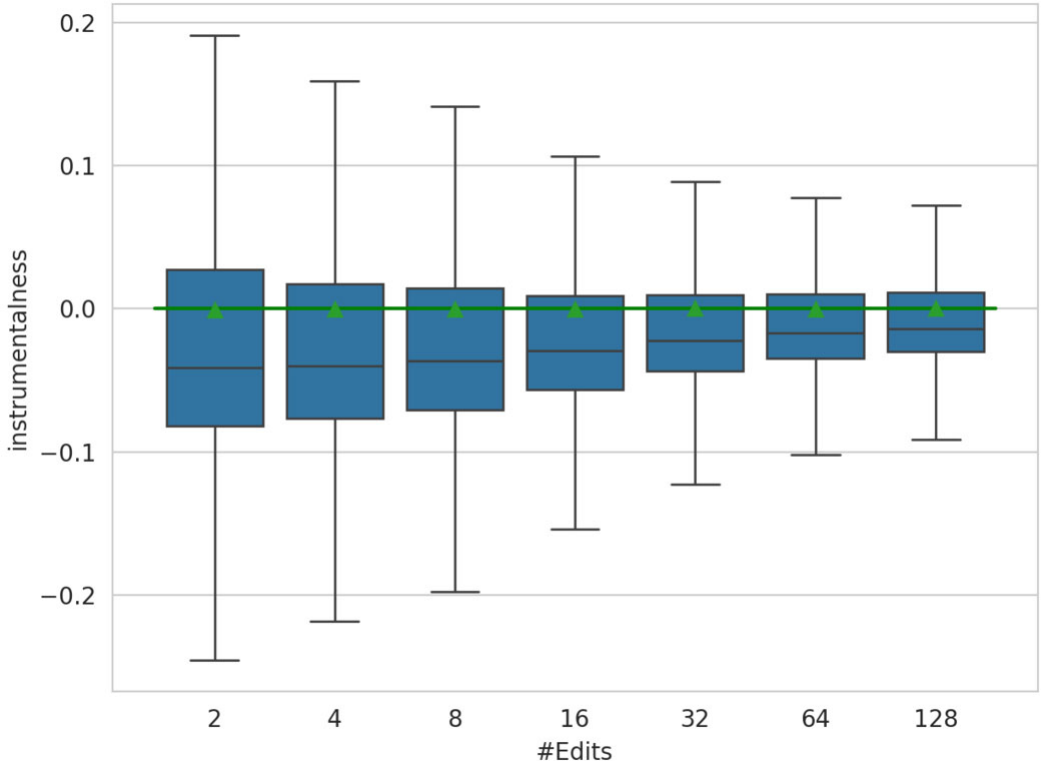
Coherence Whisker Plots of Audio Feature Instrumentalness and Attribute Number of Edits on Shuffled Playlists. The plots are grouped by applying a base-2 logarithm to the number of playlist editing sessions and rounding down to an integer

Coherence analysis conducted on features that are not normally distributed will naturally exhibit a diverging median from zero, depending on the sequence length. As a result, playlist length and all attributes that correlate positively with the playlist length, will inherently bias toward positive correlations in coherence when using methods that relate to the median. This effect is visible in Fig. 7, which illustrates Whisker plots on coherence for shuffled playlists with varying numbers of edits on the feature *instrumentalness*. While the mean remains around zero, playlists with fewer edits show negative coherence values in the median. As the number of edits increases, the median coherence values gradually rise towards zero, as heavily edited playlists tend to contain more tracks. Consequently, rank-based methods, e.g., Spearman’s Rho and Kendall’s Tau, which are outlier-robust, detect the trend represented by the median and deliver significant correlation results between playlist edits and *instrumentalness* even though the tracks of the playlists are shuffled. Notably, the effect will influence coherence results on playlists that are not shuffled as well. In contrast, Pearson correlation and the mathematical equivalent for dichotomous variables (point-biserial correlation), which are not an outlier-robust method, returns correlations around zero, justifying its usage for our initial assessment in the experimental setup.

#### Significance checks on large datasets

Significance checks (t-tests) of Pearson correlation assume a normal distribution. While this assumption is controversially discussed, researchers have found that it can be relaxed for large datasets [58]. Furthermore, short playlists are filtered out from the dataset to ensure that the resulting coherence distribution more closely approximates normality (cf. Sect. 5.2), even when the features follow a skewed or multimodal distribution. To provide a complete picture, significance checks for correlation experiments are presented alongside the primary significance results from causal inference.

#### Implications on experiments

Potential biases may arise when analyzing variance-based measurements, e.g., coherence and diversity, dependent on the applied method. In our scenario, Pearson and point-biserial correlation closely approximates the expected value of zero for shuffled data compared to other correlation methods. To enhance the accuracy of significance tests and ensure coherence results are less variable, short playlists are filtered out. This filtering allows Pearson and point-biserial correlations to perform more reliably by leveraging longer sequences, i.e., the number of tracks within each playlist. Furthermore, correlation and causal inference experiments (cf. Sect. 5.1) are double-checked for empirical soundness by repeating the experiments on shuffled playlists. For more insights and the mathematical reasoning behind the observed behavior, the reader is referred to the work by Kwak on the Central Limit Theorem [59].

### Tonality distance

**Figure 8:**
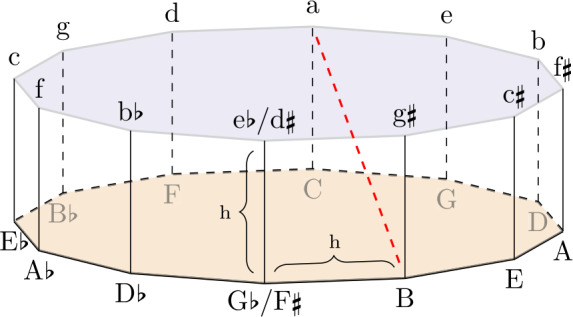
Visualization of the Tonality Distance in 3-Dimensional Euclidean Space. Major keys (upper-case letters) are positioned on the bottom polygon, and minor keys (lower-case letters) on the top. ♯ refers to sharps, ♭ to flats. The dashed red line illustrates the tonality distance between the minor key ‘a’ and major key ‘B’

To measure coherence in terms of tonality, the audio features *key* and *mode* are combined to create a distance function, as proposed by [35]. The keys are arranged according to the music-theoretical concept of the circle of fifths on a two-dimensional polygon with twelve edges and a circumradius of one. A third dimension is added to represent the mode (major or minor) of the track. The result is a prism with a height equal to the distance between two neighboring keys, $h=2 \sin (\pi / 12)$. Major keys are placed on the bottom and minor keys on top, as shown in Fig. 8.

By mapping the key and mode of a track in this three-dimensional space, the tonality distances between two tracks can be measured using the Euclidean distance between their respective coordinates. This distance metric substitutes *d* from Equation (9) to establish a coherence formula for tonality.

### Artist distance

To extend the coherence measurements beyond auditory features, artist information is included in the analysis to view coherence from a metadata perspective. The analysis focuses on the extent to which users decide to repeat or chain similar artists together to achieve a coherent listening experience. As highlighted in Sect. 3.4, each track in the MPD is queried to retrieve all featured and main performers involved in the recording. To measure coherence based on artist information, a distance metric between two tracks *k* and *l*, i.e., the distance between the artist sets $A_{k}$ and $A_{l}$, needs to be established.

First, a sparse playlist-artist matrix *M* is created, where each row *i* represents a playlist and each column represents an artist *j*. Each entry $m_{ij}$ of matrix *M* is initialized with the number of times artist *j* is involved across all tracks of playlist *i*. Similar to common text processing in information retrieval, matrix *M* can also be seen as a document-term matrix, where playlists represent documents and artists represent terms. To reduce the bias of popular artists, TF-IDF weighting, defined as $tf(t, d)/idf(t, D)$ using Equation (12), is applied to all elements of matrix *M*. Here, $tf(t, d)$ represents the frequency of artist *t* appearing in playlist *d*, normalized by the frequency of the most common artist in *d*. The inverse document frequency ($idf(t, D)$) is calculated as the natural logarithm of the total number of distinct artists in the dataset (i.e., the number of columns in *M*) divided by the number of playlists that contain artist *t*.

12 $$ tf(t, d) = \frac{f_{t,d}}{max\{f_{t^{\prime },d} : t^{\prime } \in d\}} \qquad idf(t, D) = \log{\frac{N}{|\{d: d \in D \wedge t \in d\}|}} $$

The distance between two artists *a* and *b* is calculated using the cosine distances between the respective columns of matrix *M*, denoted as $\mathbf{m}_{a}$ and $\mathbf{m}_{b}$. The distance between two artist sets $A_{k}$ and $A_{l}$ is then determined by averaging the cosine distances of all possible pairwise combinations of individual artists from the two sets:

13 $$ d_{\text{artists}}(A_{i}, A_{j}) = \frac{1}{\|A_{k}\| \| A_{l}\|} \sum _{a \in A_{k}} \sum _{b \in A_{l}} 1 - \frac{\mathbf{m}_{a} \cdot \mathbf{m}_{b}}{\|\mathbf{m}_{a}\| \|\mathbf{m}_{b}\|} $$

After establishing the distance metric, coherence formula coh (cf. Equation (9)) can be applied to sets of artists by substituting $d(x_{i}, x_{i+1})$ with $d_{\text{artists}}(A_{i}, A_{i+1})$ and $d(x_{i}, x_{j})$ with $d_{\text{artists}}(A_{i}, A_{j})$, where $A_{i}$ refers to the set of artists involved in the creation of the track at position *i* for the playlist under analysis.

## Experimental setup

The following sections cover the strategies, i.e., correlation analysis, and causal inference, that lead to the evaluation of the extent to which the four playlist attributes influence the 11 coherence features. Section 5.1 outlines the correlation analysis and continues with a detailed description of the causal inference approach. Before these strategies can be applied, playlists that might reduce the expressive power of the analysis are filtered and categorized, as explained in Sect. 5.2.

Although correlation and causal inference are applied consistently across all research questions, variations exist in the correlation methods used, the scaling of attribute values, and the ranges selected for categorization to perform causal inference. Table 2 provides an overview of these differences, along with the research questions and their corresponding playlist attributes, which are described in more detail in the following sections.

**Table 2:** Experimental Setup Overview

	Attribute	Correlation	Scaling	Causal inference		
				*c*	$t_{1}$	$t_{2}$
RQ1	playlist length	Pearson	log	11 − 40	41 − 100	101 − 250
RQ2	track popularity	Pearson	none	0 − 6.35	6.36 − 7.80	7.81 − 10.3
RQ3	num_edits	Pearson	log	1 − 4	5 − 40	41 − 201
RQ4	collaborative	point-biserial	none	PP	CP	

The first and second rows link each research question to the respective attribute used in the analysis. The “Scaling” column describes the transformations applied to attribute values before performing one of the two correlation methods and the causal inference analysis. The remaining columns outline the ranges used to classify playlists under analysis into control, first treatment, or second treatment groups based on their attribute values prior to transformation. Notably, the binary collaborative attribute has only two categories: personal playlist (PP) and collaborative playlist (CP).

### Correlation & causal inference analysis

#### Continuous attribute scaling

First, two of the continuous playlist attributes to be analyzed, i.e., playlist length and the number of edits, are scaled by the natural logarithm to adjust their relative impact in the analysis. This logarithmic scaling helps ensure that smaller absolute differences are given more emphasis when they are more meaningful. For instance, the difference between a playlist with 25 tracks and one with 50 tracks is more significant (a doubling) than the difference between a playlist with 200 tracks and one with 225 tracks, even though both pairs differ by 25 tracks. Additionally, visual inspection revealed that coherence trends become more linear after applying logarithmic scaling, improving the applicability of the data for both correlation and causal inference analysis. It is important to note that we did not further transform the track popularity attribute, as logarithmic scaling is already incorporated in the process of measuring the popularity of individual tracks (cf. Sect. 3.2).

#### Correlation analysis

Pearson correlation is computed for each pairing of continuous attributes and individual coherence features to provide an initial assessment of linear relationships. For the binary collaborative attribute, point-biserial correlation is used. As highlighted in Sect. 4.5, preliminary experiments showed that Pearson and point-biserial correlation delivers more accurate results in comparison to other popular correlation methods, reasoning its usage. Performing correlation experiments is advantageous to observe the strength and direction of the linear relationship between individual attributes and a specific coherence feature. This reveals how changes in one attribute are associated with changes in coherence, providing insights into potential patterns or trends. However, correlation does not imply causation, so it only identifies associations, not the underlying reasons for them. For example, as highlighted later, it is possible to quantify that an increase in the number of edits correlates with a decrease in coherence. However, this relationship may be influenced by playlist length or track popularity, which is not directly accounted for in the correlation analysis, making it hard to understand if number of edits is the main reason for the observed decrease in coherence. In contrast, causal experiments rely on manually assigned categories, which introduces a potential source of error. Correlation analysis complements this by offering additional robustness, as attributes are analyzed and compared systematically without user intervention, making the results more reliable.

#### Causal inference

The results in Table 3 indicate that the number of edits positively correlates with playlist length, and the collaborative attribute shows a slight interrelation with playlist length, number of edits, and track popularity.^8^ To isolate the impact of each attribute on playlist coherence, causal inference is used as a statistical approach designed to estimate the effect of a specific variable (often referred to as a *treatment*) on an outcome, while accounting for the influence of other variables (confounding variables). Although the correlation values between the attributes are mostly small or insignificant, the effects of combinations of confounding attributes could influence the coherence results in ways not detectable by correlation analysis alone. Furthermore, significance checks in causal inference experiments are more accurate compared to those in Pearson and point-biserial correlation methods, as the latter typically assume a normal distribution of data. While this assumption can be relaxed in large datasets (cf. Sect. 4.5), causal inference methods are inherently designed to handle deviations from normality. This makes their significance checks more robust and reliable, especially under diverse data conditions.

**Table 3:**
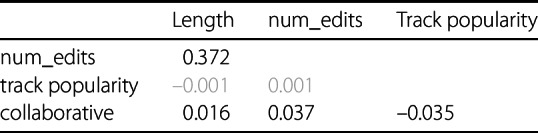
Correlation Matrix of Playlist Attributes

Correlations between playlist length, number of edits, and track popularity are calculated using Kendall’s Tau. Correlations referring to the binary collaborative playlist property use point-biserial correlation. Non-significant correlations with a p-value above 0.005 are displayed as gray numbers.

In our experiments, all three attributes not under analysis are included as confounding factors to estimate the effects on the treatment variable (the attribute under analysis) more accurately and to account for possible complex interactions.

#### Categorization between treatment and control group

To evaluate the impact of individual attributes on coherence using causal inference, playlists are categorized into three distinct groups based on the magnitude of the attribute under investigation. For example, playlists are divided into groups of static, dynamic, and highly dynamic playlists according to their number of edits. These groups serve as proxies to explore whether and how coherence changes when static playlists are modified/treated over multiple editing sessions. Sticking to the terminology, static playlists serve as the control group (*c*), while dynamic and highly dynamic playlists represent the treatment groups ($t_{1}$ and $t_{2}$, respectively). This leads to two experiments: one comparing the control group with the treatment group of dynamic playlists (), and another comparing the control group with the treatment group of highly dynamic playlists (). The reason why the experiment is performed on two treatment groups is to provide better insights into the extent to which varying intensities of the treatment group influence coherence. For the binary collaborative attribute, no categorization is performed, and personal playlists are compared only once against collaborative playlists, cf. Sect. 5.2 for more details on the exact splitting procedure for all attributes.

#### Doubly-robust Horvitz-Thompson

In our analysis, the doubly-robust version of the Horvitz-Thompson weighting estimator [60] is used. This estimator adjusts for the fact that different playlists have different probabilities of being included in a particular treatment or control group. These probabilities, known as propensity scores, are estimated based on observable characteristics of the playlists. The weighting process ensures that the comparisons between groups account for these probabilities, making the results less biased by the natural imbalance in the dataset. For example, there are typically more personal playlists than collaborative ones. Without proper adjustment, this imbalance could skew the analysis by overrepresenting the characteristics of personal playlists in the results. By applying the weighting process, the estimator ensures that both personal and collaborative playlists are fairly represented in the comparisons.

In addition to propensity score model, a regression model is used to predict the coherence feature based on the playlist attributes. This model estimates what the coherence feature would be if the playlist had been in a different group, allowing for more accurate comparisons, both in terms of effect estimation and significance tests. The doubly-robust version of Horvitz-Thompson weighting estimator remains valid (produces unbiased estimates of the treatment effect) as long as one of the two models, either the propensity score model or the outcome regression model, is correctly specified.

The primary metric for the causal inference experiments is the average treatment effect (ATE), which estimates the average difference in playlist coherence between categories, assuming that all other factors are held constant.

#### Bonferroni correction for significance tests

In total, 121 causal experiments and significance tests are performed. However, without adapting the threshold of the *p*-value, the probability of a Type I error (i.e., incorrectly rejecting a null hypothesis) increases due to the execution of multiple experiments in parallel. To address this, Bonferroni correction is applied, meaning that experiments need to return a p-value below $0.05 / 121$. Furthermore, to validate the robustness of our coherence analysis, the experiments are repeated with the same setup but with shuffled playlist tracks. The results of these experiments are provided in the supplemental material.

### Pre-processing

**Figure 9:**
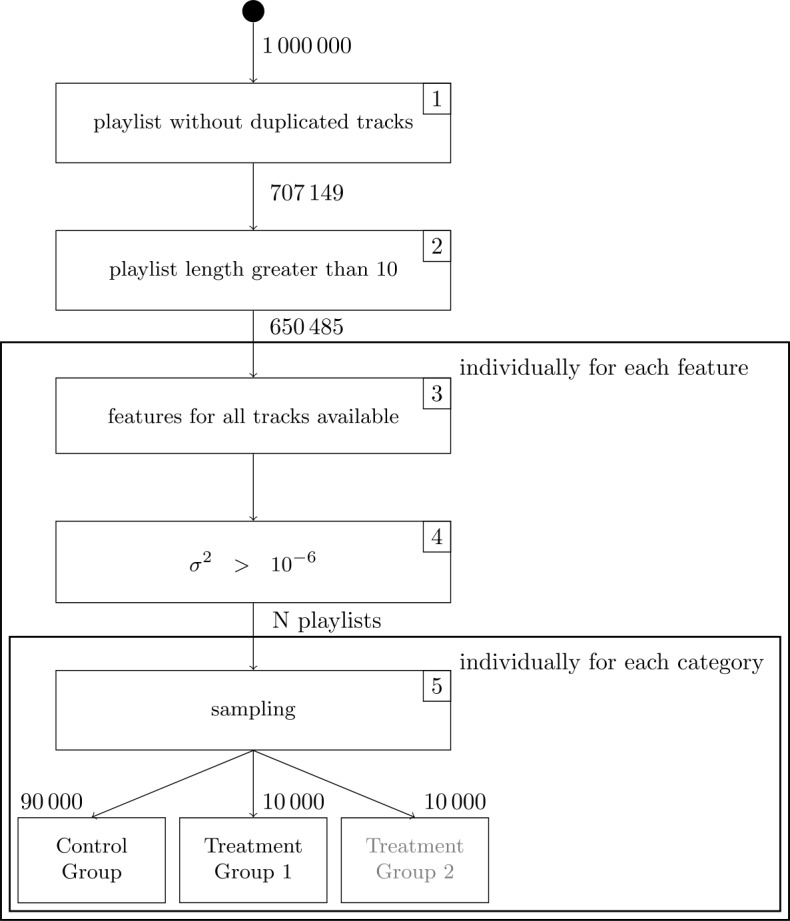
Flowchart Representation of the Filtering Pipeline. Each step (1–5) contains a constraint required to be satisfied. The numbers between the nodes indicate the number of remaining playlists after applying each constraint. Step 3 and 4 are executed individually for each feature under analysis, leading to different numbers of playlists (cf. column N in Table 4). Sampling in Step 5 is only applied for causal inference experiments. Treatment group 2 is highlighted in gray, as experiments on the collaborative attribute do not have a second treatment group

The following section describes the filtering steps applied to improve the quality of the data and the splitting procedure used to build the categories for causal inference. For orientation, a graphical representation of the pipeline (steps 1 to 5) is provided in Fig. 9.

#### Filtering of playlist with duplicate tracks

Analyzing the dataset showed that 29% of all playlists contain at least one track more than once. It is important to distinguish between the terms *song* and *track*. A song refers to a unique piece of music identified by its melody, lyrics, and harmony, while a track refers to a specific recording of a song. Our first observation is that duplicated tracks within playlists bias the analysis towards more coherent results, especially if the same track has been added multiple times in a row. Since repeating tracks have a sequential variance of zero, outliers are more likely to occur. Secondly, some music streaming services, such as *Deezer*,^9^ do not allow playlists with duplicated tracks, and others, like Spotify since 2023 [61], only allow adding two same tracks through workarounds. Due to these reasons, in filtering Step 1 playlists with duplicate tracks are filtered out. Playlists containing different tracks that refer to the same song are not affected by this filtering strategy, as this use case is generally accepted by all music streaming providers.

#### Filtering of short and invalid playlists

In Step 2, playlists with 10 or fewer tracks are excluded from the analysis to reduce the spread of the coherence results that naturally appear with fewer tracks (cf. Sect. 4.5). Furthermore, the API did not return audio features for 0.025% of all tracks. In the rare cases where a playlist contains a track for which the current audio feature under analysis is missing, the playlist is not included in the respective experiment (Step 3).

In Step 4, playlists for which $\sigma ^{2}$ is smaller than 10^−6^ are removed from the analysis of the feature currently being evaluated for stability reasons. This is necessary, because $\sigma ^{2}$ acts as a denominator in the coherence formula. Notably, this constraint affects only a few playlists and primarily impacts audio features that are heavily skewed, e.g., *instrumentalness* and to a lesser extent *speechiness*, cf. Table 4 for the exact number of remaining playlists after this filtering step. For the correlation analysis, all remaining playlists after applying these filtering strategies are used.

#### Categorization for causal inference

The primary analysis technique of this study relies on causal inference, which requires the playlist property under analysis to be discretized into categories. To answer to what extent *playlist length* influences the coherence (RQ1), playlists are split according to the number of tracks. Short playlists with a length of 11–40 tracks are designated as the control group (*c*), medium-sized playlists with 41–100 tracks as treatment group 1 ($t_{1}$), and long playlists with 101–250 tracks as treatment group 2 ($t_{2}$). As described in Sect. 5.1, ATE experiments are conducted twice: first, comparing the control group with treatment group 1 (), and second, comparing the same control group with treatment group 2 (). Generally, the intervals for all continuous categories were chosen to balance the number of playlists within each category while considering the observation that attributes and coherence tend to follow a logarithmic relationship.

To evaluate the effect of *track popularity* on coherence (RQ2), playlists are categorized into equally sized groups due to the shape of the distribution in the MPD, cf. Fig. 4. The lower third of playlists, containing less popular music, form the control group (*c*), which is compared against the treatment groups containing some popular music ($t_{1}$) and a lot of popular music ($t_{2}$).

To investigate whether there is a difference in coherence between dynamic and static playlists (RQ3), the *number of edits* on a playlist a user performs within a session is analyzed. As the number of playlist edits shows a pronounced long tail distribution (cf. Fig. 2), the dataset is split into groups with edit counts of 1–4, 5–40, and 41–201. Similar to playlist length and track popularity, the static playlist group (1–4 edits counts) serves as the control group (*c*), while the other two are assigned as the treatment groups, referring to dynamic ($t_{1}$) and highly dynamic playlists ($t_{2}$).

The last playlist property for coherence analysis observes potential coherence differences between *collaborative and personal playlists* (RQ4). Since this property is a predefined binary category, causal inference is applied only once, with collaborative playlists as the treatment group and personal playlists with a single curator as the control group.

#### Balancing sample size

To make significance checks on causal inference comparable across all playlist attributes, the sample sizes must be equal within the control groups and within the treatment groups. After Step 4, only 12,839 collaborative playlists remain for the *instrumentalness* feature, limiting the treatment group size for all experiments. Consequently, in Step 5, all treatment groups are capped at 10,000 playlists. For the control groups, including more samples (i.e., 90,000 playlists) allow the Horvitz-Thompson weighting method to produce more reliable estimates. In total, all causal inference experiments have exactly 100,000 playlists available for analysis, a sufficient amount as statistical significance tests become very powerful with this sample size.

## Results

**Figure 10:**
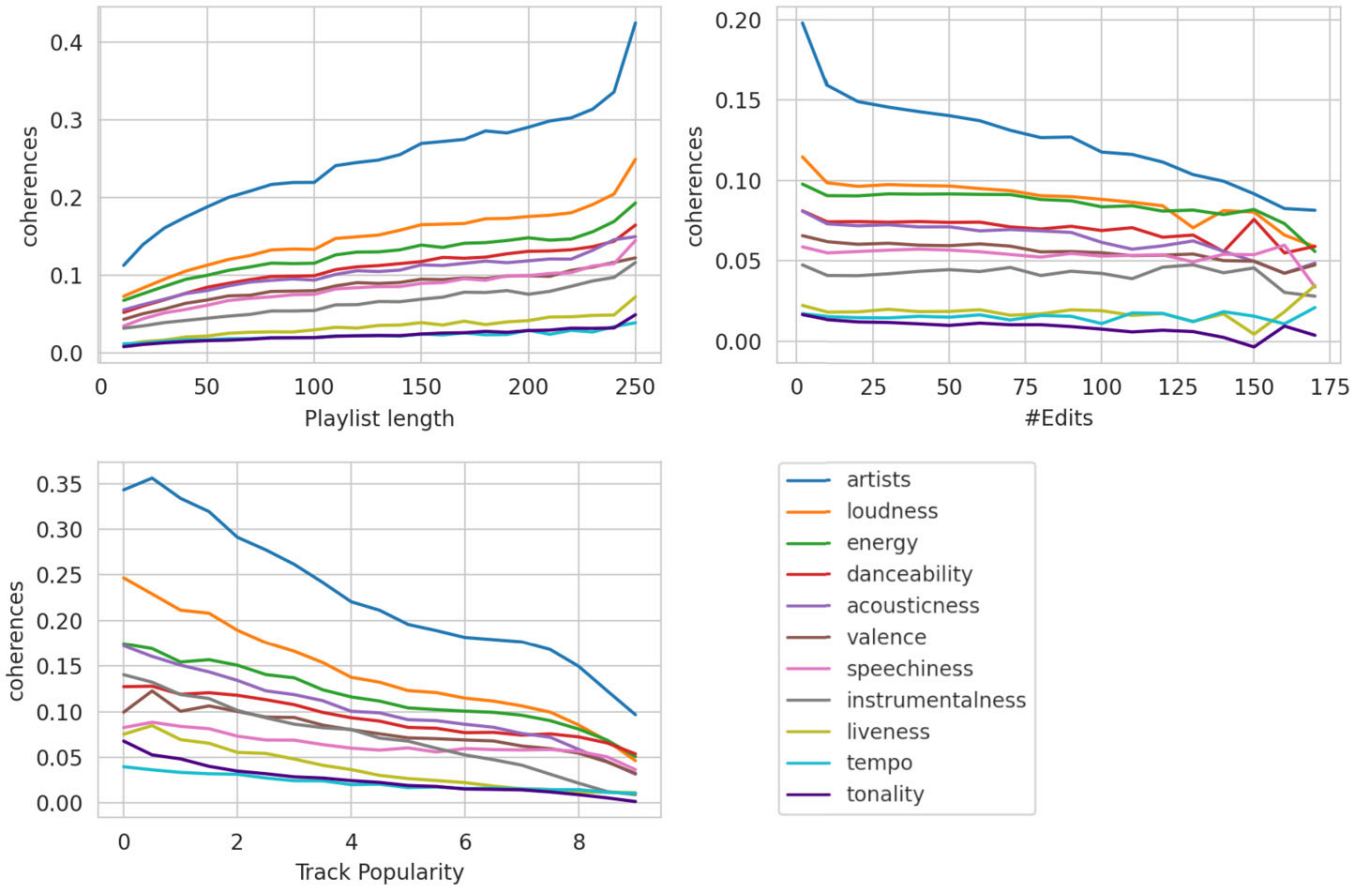
Coherence Trends Across Continuous Attributes. These three plots depict the relationship between playlist coherence (y-axis) and the three continuous playlist attributes (x-axis). Playlist length and the number of edits are shown before logarithmic scaling and are grouped into bins of 10 for visual clarity. Track popularity is grouped into bins of 0.5

**Figure 11:**
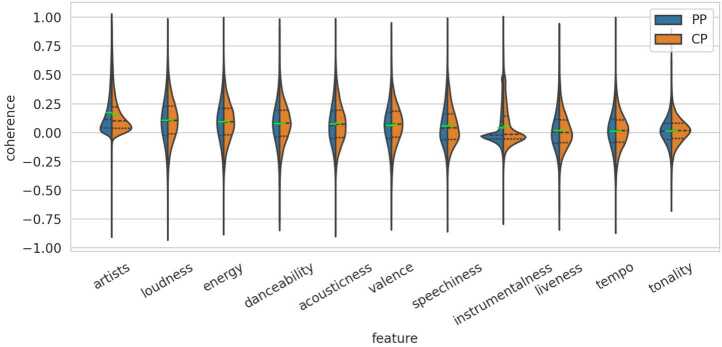
Violin Plot Comparing Personal Playlists (PPs) and Collaborative Playlists (CPs). The violin plots illustrate the distribution of coherence values across different features. The left side of each plot (blue) represents the distribution of PPs, while the right side (orange) represents the distribution of CPs. The green bars indicate the mean values, and the dashed lines mark the quartiles

In the following, the coherence analysis is presented using four sources. Figures 10 and 11 visualize the coherence trends for the three continuous attributes and the collaborative attribute, respectively. The main results of the correlation and causal inference experiments are detailed in Table 4, which also includes the average coherence values for each feature and category after subdividing the playlists according to their attributes (processing Step 4).

**Table 4:**
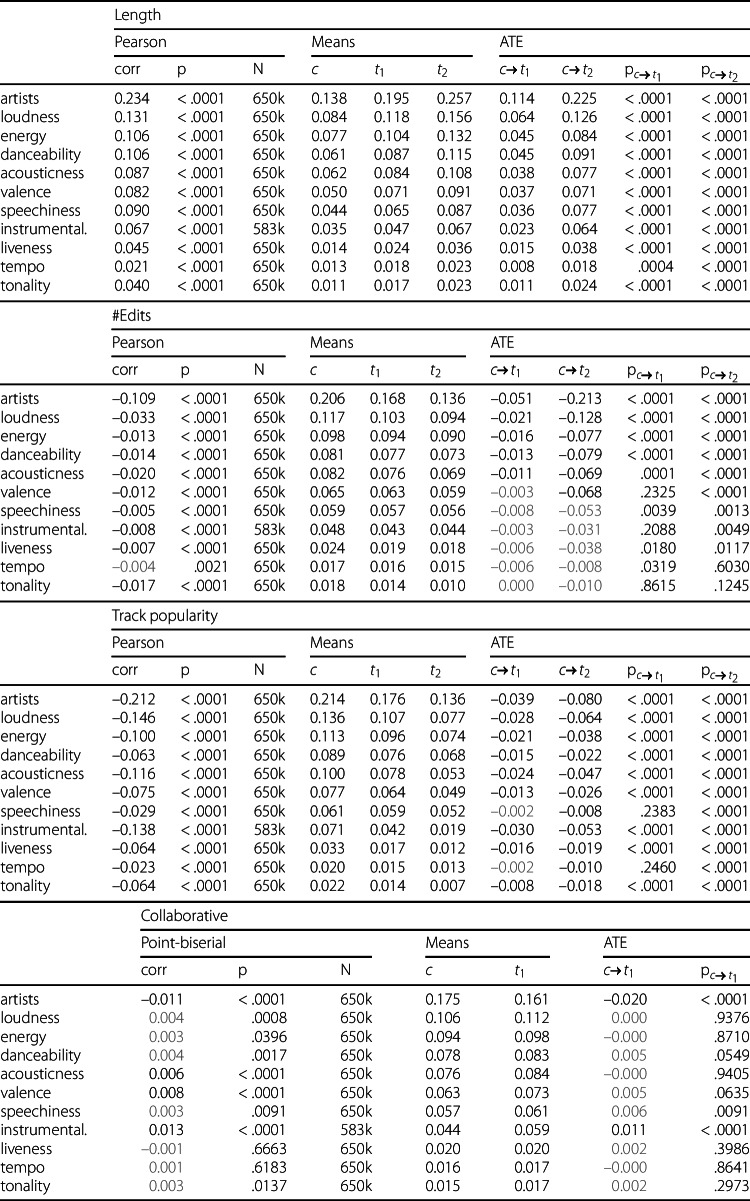
Results of Correlation and Causal Inference Analysis

Column *corr* displays the Pearson and point-biserial correlation measured over all playlists not affected by filtering. *Means* displays the average coherence of the three categories after processing Step 4, e.g., *c* refers to low playlist length, #edits, track popularity, and PPs. The two *ATE* columns describe the average treatment effect between the control group and treatment group 1 and between the control group and treatment group 2. Notably, there are only two groups for calculating means and one ATE column for the binary collaborative playlist property. Correlation and ATE scores without significance are highlighted with gray numbers.

### Correlation results

The results displayed in Fig. 10 indicate that, on average, users tend to curate coherent playlists, as nearly all lines in the graph remain above zero. The only exception is the tonality feature at 160 edits, likely due to noise caused by the limited number of playlists with such a high edit count. This observation is further supported by the mean values in Table 4. Additionally, the strength of coherence varies across different features, both overall and for the specific attributes analyzed.

#### Playlist length

There is a positive correlation between the playlist length and coherence features, meaning that long playlists tend to be more coherent than short ones. This effect is detectable throughout all features, with all p-values below 0.001. Notably, the correlation value of 0.234 between playlist length and artist coherence represents the strongest interrelation among all the correlation experiments.

#### Number of edits

For the playlist attribute number of edits, a negative correlation is observed. This suggests that playlists become less coherent the more often they are modified. For all analyzed features, it can be observed that the coherence values for the number of edits attribute are relatively low in terms of absolute numbers, with the audio features being equal to or below 0.033 and artist coherence correlating less than half as much as the playlist length and track popularity attributes. Additionally, there is no statistical significance measurable for the coherence feature *tempo*.

#### Track popularity

For the playlist attribute track popularity, a negative correlation is observed as well, indicating that playlists that contain popular music are less coherent than playlists with unpopular music. Track popularity has similar strong correlation effects in absolute numbers than the playlist length attribute, with a majority of audio features showing even higher numbers, i.e., *loudness*, *acousticness*, *instrumentalness*, *liveness*, *tempo*, and *tonality*. Similar to the playlist length experiments, all p-values are below 0.001 as well.

#### Collaborative

The point-biserial correlation analysis on collaborative playlists yields values close to zero, with most features failing the significance checks. Only *artists*, *acousticness*, *valence*, and *instrumentalness* show significant relevance. Among these, artist coherence, followed by *instrumentalness*, appears to have the largest impact, which can also be seen in Fig. 11, where the green bars are notably displaced. This observation is further supported by the differences between the mean values of personal playlists (*c*) and collaborative playlists ($t_{1}$) in Table 4. Interestingly, the artist coherence feature correlates negatively, indicating that artists are more often repeated or followed by similar artists in personal playlists. In contrast, all significant audio features show a positive correlation, suggesting that tracks within collaborative playlists are slightly more coherent in terms of audio features compared to personal playlists.

### Causal inference results

#### Playlist length

Switching to the causal inference results, a generally similar picture emerges for playlist length. The ATE coherence values show that medium-sized playlists with 41–100 tracks are more coherent than short playlists with 11–40 tracks (). The trend is consistently around twice as large when short playlists are compared against long playlists with 101–250 tracks across all features (). Since all p-values are below 0.001 with the only exception of *tempo* having a value around 0.004 for  (which is still significant), playlist length appears to be an influencing factor on its own for playlist coherence. Additionally, the ATE values of  and  are larger than the direct differences between the means of *c* and $t_{1}$, as well as between *c* and $t_{2}$. This suggests that some other attributes may reduce the coherence effect associated with playlist length.

#### Number of edits

For the number of edits attribute, the negative correlation is also reflected in the ATE values. While the absolute correlation values for this attribute are relatively small compared to those for playlist length and track popularity, the ATE values tell a different story, particularly when comparing static playlists with 1–4 edits to highly dynamic playlists with 41–201 edits (). For instance, *artists*, *loudness*, and other significant features exhibit similar changes in coherence when considering their absolute values for , as observed with playlist length.

Together with the observation that the number of edits correlates with the playlist length ($\text{corr}=0.372$), a complete picture emerges. High editing numbers are more likely to be found in long playlists, which, as established, are more coherent than short playlists. However, editing a playlist over multiple sessions reduces the coherence values of the playlists. Therefore, the number of edits has a similar impact on coherence as playlist length when considered independently of other confounding variables in highly dynamic playlists. It is likely that the number of edits is the primary factor contributing to the previously mentioned reduction in coherence for playlist length compared to the ATE values. Additionally, we notice that the ATE results for dynamic playlists are not significant for some features, i.e., *speechiness*, *instrumentalness*, *liveness*, *tempo*, and *tonality*, a topic that is further discussed in Sect. 6.3.

#### Track popularity

In contrast to the number of edits attribute, track popularity shows similar correlation values for coherence relative to playlist length, but this observation does not translate to the ATE values which are two to three times smaller by comparison. Features that usually exhibit larger coherence changes, e.g., *energy*, *loudness*, and *artists*, do not reach the ATE values observed for the number of edits and playlist length. Comparing the differences between the mean (*c*, $t_{1}$, $t_{2}$) and the ATE values demonstrates that the ATE values are only slightly larger (in absolute terms), indicating that track popularity is relatively unaffected by the other playlist attributes. This is further supported by the correlation results in Table 3, which show no significant correlations between track popularity and the other two continuous attributes, i.e., playlist length and number of edits. The collaborative attribute shows slight interrelations with track popularity, but does not influence coherence enough (cf. next paragraph) to make a difference for the ATE values of track popularity. Nevertheless, most ATE results are significant, except for *tempo* and *speechiness* in the  comparison. This suggests that while the popularity of tracks within playlists might not negatively affect coherence as much as the number of edits does, the influence is still measurable nearly across all features.

#### Collaborative

The ATE values for collaborative playlists largely confirm the irrelevance of this attribute, except for the *artist* and *instrumentalness* features. While a comparison of the mean values between personal and collaborative playlists shows a slight increase in coherence for certain audio features, this trend diminishes when causal inference accounts for confounding factors, resulting in ATE values close to zero and non-significance for all audio features except *instrumentalness*. In contrast, causal inference reveals a smaller ATE value for the *artist* feature ($c \rightarrow t_{1} = -0.020$) compared to the difference in mean values ($t_{1} - c = -0.015$), indicating that collaborative playlists are less coherent in terms of artist consistency when confounding factors are removed. Nevertheless, the subtle differences observed in both significant features call into question the overall relevance of this attribute.

### Discussion

#### Playlist length

Analyzing the correlation and ATE values, showed that long playlists tend to be more coherent than short ones. We believe that this observation is primarily due to the way users create long playlists. The correlation results between attributes (cf. Table 3) support the claim that longer playlists are created in multiple editing sessions.

We hypothesize that during a single editing session, a user adds multiple tracks that are similar to each other, reflecting the user’s current mood, context, or general objective of the playlist. As time passes between editing sessions, the user’s idea of what the playlist should represent may evolve, along with their mood or even music taste.^10^ Tracks added in later sessions might be consistent with each other, but may not align perfectly with the playlist’s original theme. Thus, the time elapsed between the playlist’s creation and the addition of recent tracks is likely a major factor in playlist coherence. Unfortunately, the dataset only includes the time of the last modification, not the creation date, leaving this hypothesis open for future investigation.

#### Number of edits

When presenting the results for the number of edits, the focus was on the ATE results between static and highly dynamic playlists. Although the categorization with editing groups of 1–4, 5–40, and 41–201 are rather unbalanced, we expected the ATE to follow a ratio comparable to the results for the playlist length, i.e., . However, the coherence difference between static and dynamic playlists () is approximately four times smaller than that between static and highly dynamic playlists ().

We hypothesize that the decrease in coherence is not primarily due to the addition of tracks across multiple sessions but rather due to other modification actions, i.e., reordering, removing, or replacing tracks. Supported by the findings in [2], we believe users initially focus on filling playlists with fitting tracks, resulting in coherent playlists. Over time, as the playlist becomes saturated, the curator likely transitions to an optimization phase, where repeating artists and similar songs are interrupted by reordering, and disliked tracks are replaced or deleted. This optimization phase begins probably after several editing sessions and peaks in highly dynamic playlists, explaining the unexpected imbalance between the two causal inference experiments.

Additionally, the ATE results for the number of edits analysis failed to show significance for some audio features. However, *speechiness*, *instrumentalness*, and *liveness* exhibit ATE values below −0.03 for , which are larger in absolute terms than some of the significant results observed for track popularity. The strong correlation between playlist length and the number of edits likely makes it challenging for causal inference to identify short playlists with a high number of edits. This difficulty may lead to larger p-values for these features, reducing the statistical significance of the results.

#### Track popularity

The results for the track popularity attribute reveal that playlists with popular tracks are less coherent than those with unpopular music, which aligns with our expectations. When users focus on a single quality criterion (in this case, adding popular tracks) other quality criteria tend to be neglected. This type of heuristic simplification is commonly observed in various research fields that study user decision-making processes [63]. However, we are surprised to find that focusing on popular tracks did not affect the length of playlist, as evidenced by both the attribute correlation results in Table 3 and the ATE results for track popularity.

#### Collaborative

Finally, we observed that collaborative and personal playlists show only small differences in point-biserial correlation and no significant differences in coherence according to the causal inference experiments, except for the *artist* and *instrumentalness* features. These results are surprising, as previous research has reported that users behave differently in the curation process when collaborators are involved [25, 40]. In particular, curators may feel uncomfortable deleting tracks added by others and tend to add tracks that are likely to be enjoyed by everyone, often leading to more popular track choices. Additionally, the theme of collaborative playlists is often more clearly defined so that all participants understand which tracks are appropriate. The situational context in which collaborative playlists are created can also vary, such as serving as a queue during a party.

We hypnotize that while playlists in the dataset are marked as collaborative, this does not necessarily indicate active involvement from other collaborators in the creation process. As explained in Sect. 3.2, a playlist is considered collaborative if another user joins as editor. However, collaborators can remain passive without making any contributions. At the time the dataset was collected, creating collaborative playlists was a relatively new feature, which may have led to overly cautious and passive collaborators due to inexperience with this feature and its intended purpose. Table 3 supports this hypothesis, as the correlation results between the three attributes and collaborative versus personal playlists show only minor interdependencies. This finding contradicts previous research suggesting that collaborative playlists tend to fill up quickly with numerous (popular) tracks [24].

However, to validate this hypothesis, it is necessary to examine whether collaborative playlists differ in coherence from personal playlists in newer datasets. This analysis will be addressed in future work.

### Feature-related findings & discussion

In addition to the coherence results for different playlist attributes, there are general findings that apply to the observed features. Figure 10 and the mean values in Table 4 show that not all features are equally important for playlist coherence. For instance, the coherence values for the artist feature consistently reach the highest averages across all attributes (*c*, $t_{1}$, and $t_{2}$, if applicable). This suggests that users tend to sequence the same or similar artists when curating a playlist. While previous research [2] found that repeating the same artist in sequence is discouraged for creating an ideal mix, it appears users break this rule [36], possibly due to the way music platforms present tracks, such as displaying other top tracks from the same artist when searching by name or adding entire albums.

Some audio features are more relevant to coherence than others. For example, *loudness*, *energy*, *danceability*, *acousticness*, and *valence* consistently show high coherence values and are significantly correlated with the attributes playlist length, number of edits, and track popularity. In contrast, *tempo* appears to be the least important feature for coherence, as it reached the highest p-value for playlist length and failed significance tests in six out of eight cases for the other attributes. Additionally, the tonality feature seems less relevant to playlist coherence, especially concerning causal inference results, which is intriguing given that DJs often consider matching keys crucial for smooth transitions [35, 64].

One final observation from Fig. 10 is that the lines rarely intersect, indicating that strong coherence values generally correspond with the largest changes in coherence when analyzing attribute influences. The only intersections occur at the ends of the plots, where fewer playlists are available for analysis, introducing noise. An exception is the *instrumentalness* feature, which stands out in the track popularity graph with a steeper slope compared to similarly high coherence features like *speechiness* and *valence*. This may be because popular tracks are often not purely instrumental, and combined with the skewed distribution seen in Figs. 5 and 11, could result in the larger slope for track popularity. However, this hypothesis would require further analysis in future research.

## Practical application

This section introduces a greedy algorithm to demonstrate the practical applications of the findings on the relationship between coherence and playlist attributes. Related to Example 3 of the introduction (cf. Sect. 1), the algorithm aims to automatically reorder the tracks of an existing playlist to align them with the coherence scores expected based on the playlist’s attributes and our findings. The algorithm can be applied to playlists that are overly smooth and coherent, where similar tracks follow one another in sequence, or to playlists that seem randomly assembled, with abrupt transitions between tracks. Additionally, the algorithm is effective in cases where only a subset of coherence features are overly smooth while others are too random, balancing the playlist to achieve a more cohesive listening experience.

The algorithm is primarily designed to demonstrate the practical applicability of the findings. To achieve this, pragmatic choices have been made to reduce its complexity without compromising its ability to optimize the coherence of an existing playlist. Consequently, the algorithm is intentionally kept simple, featuring only a few hyperparameters and avoiding complex weighting schemes.

Section 7.1 outlines four quality targets that any playlist reordering algorithm should generally adhere to. Section 7.2 explains how the most likely appreciated coherence level for a playlist can be estimated, and how the allowable differences between transitions of subsequent tracks are determined. Certain track sequences must remain unbroken during the reordering process, as discussed in Sects. 7.3 and 7.4. During the rearrangement process, the algorithm aims to minimize two error metrics: coherence error and transition error (cf. Sects. 7.5 and 7.6, respectively). After establishing this foundational framework, Sect. 7.7 presents the algorithm, and Sect. 7.8 demonstrates its functionality with a concrete example. Finally, Sect. 7.9 discusses the algorithm’s limitations and potential areas for improvement.

### Quality targets

Independent of the proposed algorithm, there are four quality targets that should generally be considered during the rearrangement process of an existing playlist.

#### Quality Target 1

The first and last tracks of the playlist must remain in their original positions. Research has shown that the first track, and to a lesser extent the last track, hold significant meaning for the curator [5, 28]. Additionally, as previously discussed, playlists are often built gradually over time, reflecting the user’s evolving music taste [3, 7]. Consequently, the first and last tracks serve as anchors, marking where this journey began and ended.

#### Quality Target 2

For similar reasons, tracks should be reordered in close proximity to their original positions. The algorithm should prioritize swapping directly neighboring tracks over those further apart. This ensures that the overarching musical progression created by the user, spanning from the beginning to the end of the playlist, remains largely intact [2].

#### Quality Target 3

While not universally true for every playlist, certain tracks are often meant to be listened to in a specific sequence [2, 28]. These pairwise sequences must be preserved during the reordering process.

#### Quality Target 4

Careless rearrangement of tracks can result in transitions with abrupt changes in musical style. Therefore, the algorithm must evaluate the compatibility of future adjacent tracks before each rearrangement. Additionally, transitions that are too smooth should be avoided as well [16].

### Finding the target coherence & distance

To optimize a playlist, the proposed algorithm requires two estimates: the target coherence level to be achieved through rearrangement and the average preferred difference between subsequent tracks. While the primary objective is to optimize for coherence, the second estimate is required to meet Quality Target 4, which addresses the avoidance of transitions that are either too smooth or too abrupt in musical style.

As established by our findings (cf. Sect. 6.1 and 6.2), three attributes influence playlist coherence, i.e., *playlist length*, *number of edits*, and *track popularity*. In contrast, differences between collaborative and personal playlists delivered mixed results and are therefore not taken into account. To estimate the generally preferred coherence level for a playlist being optimized, a multivariate linear regression model is trained using *playlist length*, *number of edits*, and *track popularity* as input features. Additionally, logarithmic transformations of *playlist length* and *number of edits* are included as input, as these attributes exhibit partially a logarithmic relationship with coherence (cf. Sect. 5.1). This allows the regression model to balance its prediction between the original attributes and their logarithmic transformations.

To satisfy Quality Target 4, the acceptable difference for transitions between subsequent tracks is estimated as well. This is achieved by calculating the average distance between all adjacent track pairs in each playlist. For all features, the distances between two tracks are computed similarly to the coherence calculation: for continuous features, it is the absolute difference; for tonality, the distance function described in Sect. 4.6 is used; and for artists, the cosine distance is applied (Sect. 4.7). The result is the average transition difference for each playlist, which is used as target to train another multivariate linear regression model. This second model uses the same attributes as input as the model for coherence prediction, i.e., *playlist length*, *number of edits*, and *track popularity*, along with the two logarithmic transformations.

### Finding tracks that belong together

To identify track pairs that likely belong together (requirement for Quality Target 3) the dataset is analyzed using a Bayesian probabilistic modeling approach. This method estimates the probability of a track $t_{i}$ being followed by another track $t_{j}$ by counting the number of their directed transitions $n_{ij}$ and normalizing it by the total number of times $t_{i}$ is followed by any track $t_{k}$, i.e., $\sum _{k} n_{ik}$. To address the challenges of data sparsity, especially caused by the long-tail distribution of the dataset, a symmetric Dirichlet prior is applied. This prior, represented as a constant $\alpha =1e^{-5}$ in Equation (14), smooths the observed probabilities and prevents overfitting to rare co-occurrences.^11^ This ensures that transitions with limited data support do not disproportionately influence the results.

The posterior probability of a transition $t_{i} \rightarrow t_{j}$ is then computed as follows:

14 $$ P(t_{i}|t_{j}) = \frac{n_{ij} + \alpha }{\sum _{k} n_{ij} + \alpha } $$

To fulfill Quality Target 3, all subsequent track pairs $t_{i} \rightarrow t_{j}$ in the playlist being optimized are evaluated to determine if $P(t_{i}|t_{j}) > 0.7$. If this condition is met, these track pairs must remain intact and should not be separated by the algorithm. A threshold below 0.7 has been found effective for identifying tracks that are consecutive in the original album order. While track sequences from the original album are often carried over into playlists, these segments are typically the ones that benefit the most from rearrangement. By setting the threshold at 0.7, only the most prominent track sequences are preserved, preventing the entire album order from being maintained in the playlist.

### Connecting tracks that belong together

**Figure 12:**
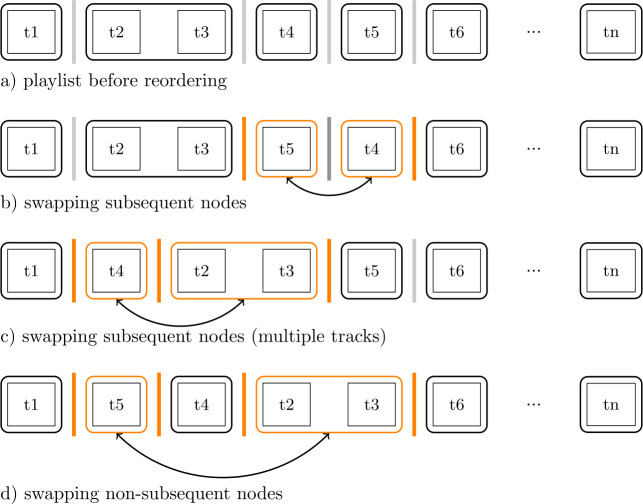
Transition Changes and Rearrangment of Tracks Under Different Conditions Using the Node System. Nodes are represented by rounded borders containing one or multiple tracks, cf. $[t_{1}]$ and $[t_{2}, t_{3}]$. Vertical bars indicate transitions between tracks. a) shows the original order of the nodes. Swapping tracks creates new transitions, highlighted in orange. b) demonstrates a swap between directly adjacent nodes, with the transition in the middle remaining unchanged (indicated by the gray bar). c) illustrates a swap involving one node containing multiple tracks (resulting in three transitions) and d) shows a swap between two nodes further apart, resulting in four transitions

To make the swapping of tracks more efficient, we implemented a system that groups one or more tracks into nodes. The reordering algorithm then operates by swapping these nodes rather than working directly with individual tracks. This abstraction is useful for handling track sequences that must remain unbroken (cf. Sect. 7.3). Tracks that belong together are grouped into the same node, enabling the algorithm to reorder both individual tracks and entire blocks of track sequences seamlessly and effectively. This concept is illustrated in Fig. 12 a), where the connection between $t_{2}$ and $t_{3}$ must remain intact and is therefore encapsulated within a single node. When the algorithm begins reordering the playlist, these two tracks are always treated as a single unit and swapped together as a whole block, as shown in Figs. 12 c) and d).

### Coherence error

The primary objective of the algorithm is to rearrange the tracks of a playlist *p* so that each coherence feature aligns as closely as possible with the general trend. To achieve this, the multivariate linear regression model from Sect. 7.2 predicts the expected coherence level $E(coh_{f})$ for a feature *f* based on the attributes of *p*. The error to be minimized is the squared difference between the expected coherence $E(coh_{f})$ and the actual coherence of the playlist in its current arrangement $coh_{f}(p)$. This error is computed for each individual feature and then summed up, as specified in Equation (15).

15 $$ \epsilon _{coh} = \sum _{f \in features} (E(coh_{f}) - coh_{f}(p))^{2} $$

### Transition error

To satisfy Quality Target 4, the transition error of subsequent tracks must be evaluated after each rearrangement. The transition error quantifies the deviation between the expected distance $E(d_{f})$ and the actual distance $d_{f}(t_{i}, t_{i+1})$ between two subsequent tracks $t_{i}$ and $t_{i+1}$ for a feature *f* and its respective distance function $d_{f}$. The expected transition distance $E(d_{f})$ is estimated using the second multivariate linear regression model based on the attributes of playlist *p* as described in Sect. 7.2. Equation (16) calculates the current transition error between two tracks (summed across all features).

16 $$ \epsilon _{trans}(i) = \sum _{f \in features} (E(d_{f}) - d_{f}(t_{i}, t_{i+1}))^{2} $$

Instead of the transition error itself, the proposed algorithm requires a measure of the improvement resulting from swapping two tracks compared to their previous order. This improvement is quantified in Equation (17), which evaluates the difference in transition error between the previous sequence $t_{i} \rightarrow t_{i+1}$ and the new sequence $t'_{i} \rightarrow t'_{i+1}$ after rearrangement.

17 $$ \Delta _{trans}(i) = \sum _{f \in features} (E(d_{f}) - d_{f}(t'_{i}, t'_{i+1}))^{2} - (E(d_{f}) - d_{f}(t_{i}, t_{i+1}))^{2} $$

Depending on whether two nodes are directly adjacent or further apart, a varying number of new track transitions must be evaluated. As illustrated in Fig. 12 b), swapping the node $[t_{4}]$ with $[t_{5}]$ creates two new transitions: one between tracks $t_{3}$ and $t_{5}$, and another between $t_{4}$ and $t_{6}$. The transition between $t_{5}$ and $t_{4}$ remains unchanged, as both nodes contain only one track and the distance functions used are symmetric, i.e., $d_{f}(t_{i}, t_{j})=d_{f}(t_{j}, t_{i})$. In contrast, when one of the nodes contains more than one track, another third transitions must be considered. For example, Fig. 12 c) demonstrates a swap between the neighboring nodes $[t_{2}, t_{3}]$ and $t_{4}$, resulting in a new transition between tracks $t_{4}$ and $t_{2}$. When nodes that are not directly adjacent are swapped, four new transitions must be considered regardless of the number of tracks within the nodes. This is shown in Fig. 12 d), where $[t_{2}, t_{3}]$ is swapped with $[t_{5}]$.

### Algorithm

This section describes the proposed procedure for rearranging tracks within playlists to improve coherence. While the general algorithm is outlined here and presented as pseudocode in Algorithm 1, the detailed implementation is available in the accompanying Git repository (cf. Section “Availability of data and materials”).

**Algorithm 1:**
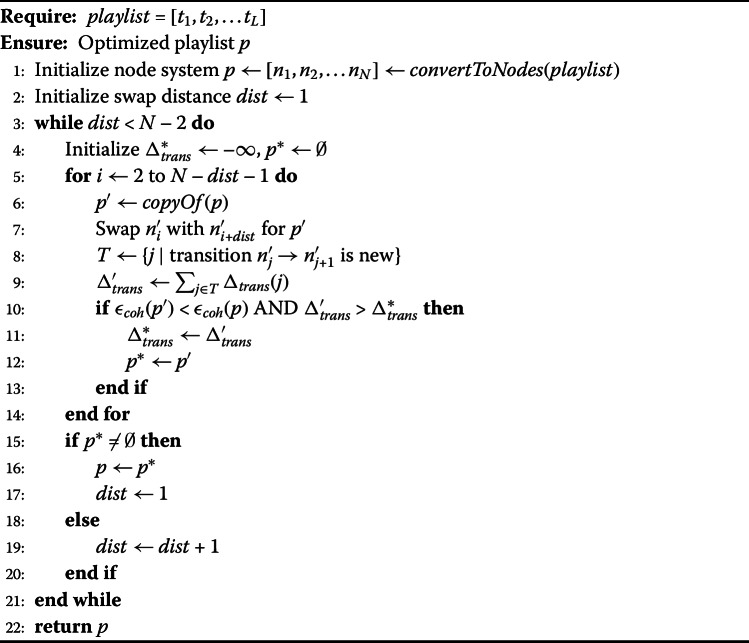
Greedy Playlist Optimization Algorithm

The algorithm begins by taking a playlist that requires optimization and initializing the node system as proposed in Sect. 7.4 (cf. Line 1 of Algorithm 1). In this step, subsequent tracks with a Bayesian probability higher than 0.7 (as per Equation (14)) are grouped into the same node to satisfy Quality Target 3. To meet Quality Target 1, the first and last tracks of the playlist remain unmodified throughout the optimization process. Additionally, to satisfy Quality Target 2, the algorithm initially only considers swapping adjacent track pairs (Line 2). This ensures that the overall musical progression, which often reflects the user’s evolving music taste over time, remains intact. If no further improvements can be achieved by swapping adjacent nodes, the algorithm increases the distance ($dist$) between nodes to consider swaps involving tracks that are further apart (Lines 18–19). However, as soon as an improvement is detected, the distance is reset to prioritize adjacent swaps again.

The algorithm continuously evaluates potential node pairs that lie a specific distance ($dist$) apart (Lines 5–7). Every iteration, a temporary playlist $p'$ is created, which replicates *p* but with the two selected nodes swapped. Among all swaps found for the current distance $dist$, the algorithm selects the $p'$ that improves the coherence error $\epsilon _{coh}(p')$ in comparison to $\epsilon _{coh}(p)$ and maximizes the transition improvement metric $\Delta _{trans}^{*}$,^12^ as defined in Equation (17). The candidate playlist $p^{*}$ that best improves both coherence and transitions is assigned as the new playlist (Lines 10–12). While the algorithm prioritizes swaps that simultaneously improve both coherence error and track transitions, it may occasionally perform swaps that only reduce coherence error at the expense of transition quality. This is intentional, as improving coherence error is the algorithm’s primary goal.

The algorithm concludes when no swaps, neither adjacent nor distant, can reduce the coherence error further. At this point, the optimized playlist *p* is returned.

### Demonstration

This section illustrates the functionality of the algorithm using a concrete example. To identify a suitable playlist for demonstration, we ranked all playlists based on the sum of their 11 coherence values. From the top 50,000 most coherent playlists, we selected one arbitrary playlist that meets the following criteria: it has a short, expressive name; contains approximately 30 to 50 tracks; and includes at least two tracks that belong together for presentation purposes. The selected playlist, identified as playlist ID 195,677 in the MPD dataset and titled *study music*, consists of 48 tracks spanning 31 albums and featuring 15 main and contributing performers. This playlist has been modified five times and has an average track popularity score of 8.142.

While the playlist exhibits high coherence values for features such as *artists* (0.731), *danceability* (0.418), and *instrumentalness* (0.369), some features exhibit zero or negative coherence values, i.e., *energy*, *acousticness*, and *speechiness*. Table 5 provides a detailed comparison of the playlist’s original coherence values alongside the target coherence values predicted by the multivariate linear regression model. The algorithm’s objective for the *study music* playlist is twofold: to reduce the excessively high coherence values for certain features and to increase coherence in features with low or negative values.

**Table 5:** Target Coherence and Playlist Coherence Before and After Reordering

Feature	Target	Original	Reordered
artists	0.194	0.731	0.199
loudness	0.108	0.181	0.112
energy	0.096	−0.003	0.099
danceability	0.084	0.418	0.090
acousticness	0.075	−0.105	0.078
valence	0.066	0.087	0.063
speechiness	0.065	−0.055	0.071
instrumentalness	0.032	0.369	0.036
liveness	0.018	0.225	0.020
tempo	0.016	0.151	0.008
tonality	0.014	0.031	0.026

The target coherence is derived from the multivariate linear regression models, predicted using the attributes of the playlist “study music” as input. The middle column displays the coherence values of the unmodified playlist, while the right column shows the coherence values after the algorithm has performed the rearrangement.

Before the reordering process begins, the playlist is transformed into the node system. In this example, each track is placed in its own node, except for the tracks at positions 39 and 40, which have a Bayesian probability of 0.713 of belonging together (cf. Sect. 7.3). Investigating the identity of both tracks reveals that both are from *Pink Floyd*’s album *Dark Side of the Moon*, specifically the first and second tracks, *Speak to Me* and *Breathe*. *Speak to Me* serves as an overture to the album, setting the stage for *Breathe*, the first fully developed track. Due to its introductory nature, *Speak to Me* is rarely listened to independently of its successor. Therefore, it is appropriately grouped with *Breathe* in a single node.

At the start of the optimization, the initial coherence error was high at 0.637. To minimize this error, the algorithm executed 95 swaps before reaching termination. As shown in the right column of Table 5, the coherence values for all features became positive and aligned closely with the target coherence values.

**Figure 13:**
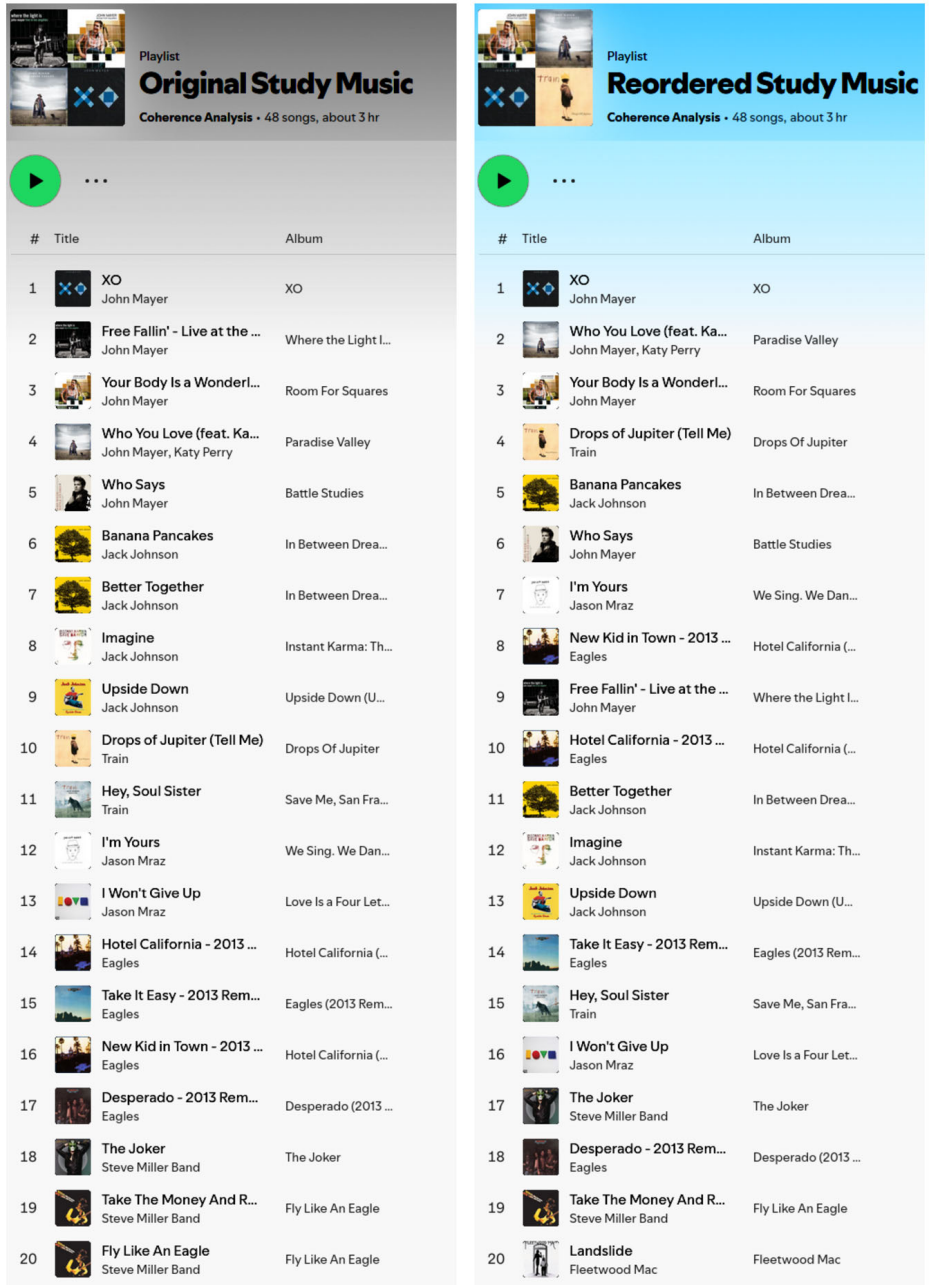
Playlist “study music” Before and After Reordering. The left panel shows the first 20 tracks of the playlist “study music” in their original order, while the right panel displays the same tracks reordered using the proposed algorithm

The original and reordered playlists for the first 20 tracks are presented in Fig. 13 and are publicable available on Spotify.^13^^,^^14^ The first observation is that sequences of repeating artists, which contributed to the high coherence value for the *artist* feature, have been significantly reduced in the reordered playlist, with only a few exceptions. Secondly, despite the high number of swaps executed during the reordering process, Quality Target 2 appears to be objectively satisfied, as most tracks remain within close proximity to their original positions.

### Limitations

While the algorithm is deterministic and effective at finding an arrangement that optimizes the target coherence, it is intentionally designed to remain simple, as its primary purpose is to demonstrate one concrete practical application of our findings. Consequently, the algorithm is relatively inefficient, as pairwise track distances are recalculated for each coherence and transition error during every iteration, rather than utilizing a precomputed lookup table. Additionally, while the calculation of distances between sets of artists (cf. Sect. 4.7) does not require hyperparameter tuning, this approach could be improved by using matrix factorization techniques, such as LightFM [65]. This would eliminate the need to compute cosine distances on very high-dimensional vectors, currently exceeding 707k dimensions in the collaborative approach.

Regarding the quality targets, in a real-world application, it may be beneficial to optimize for multiple objectives rather than focusing solely on achieving the ideal coherence level at all costs. Introducing a weighting mechanism to balance coherence, transition error, and the probability of track sequences belonging together are likely to enhance user satisfaction further.

As an additional extension, we propose the inclusion of a scalar parameter that allows users to adjust their preferred coherence level by scaling the estimated coherence values provided by the multivariate linear regression model. This would enable greater personalization and flexibility in playlist optimization.

## Conclusion and future research

In this study, we have proposed a framework for coherence measurement that focuses on the coherent ordering. This framework is applied on over 650,000 playlist to provide a comprehensive analysis of playlist coherence with respect to 11 track features by examining the effects of various playlist attributes using correlation and causal inference methods. The method offers a new perspective, independent of the type of music within playlists, and the findings will help enhance recommendation systems by reranking track suggestions to align with the coherence preferences of curators. Additionally, tools can be developed to improve the quality of existing playlists by rearranging tracks to better align coherence with general trends, as demonstrated by the simple greedy algorithm proposed in this paper. This algorithm utilizes two multivariate linear regression models: one to estimate the preferred coherence and another to identify optimal track transitions. The goal is to align the coherence values of all 11 features based on the playlist attributes analyzed in this work. While the user response to such tools remains to be evaluated in future research, statistical analyses indicate that these tools are likely to enhance user engagement. This is particularly relevant for playlists with excessive sequences of overly similar tracks or for playlists created without careful attention to track order, leading to abrupt transitions. Such tools can reduce the burden on users by automating the process of ordering tracks effectively.

Addressing *RQ**1* (To what extent does playlist length influence playlist coherence?), we observed that long playlists are on average more coherent than short ones. Differences between the correlation analysis and causal inference results indicate that the continuous addition of tracks contributes to playlist coherence. However, highly dynamic playlists for which replacing, deleting, and reordering actions predominate, show to be less coherent, which answers *RQ**3* (Do dynamic playlists, which are continuously updated, affect coherence differently than static ones?). Overall, coherence appears to be in tension between the number of edits and the playlist length. While both attributes positively correlate with each other, they affect coherence in opposite directions.

Additionally, we found that playlists featuring popular tracks are generally less coherent than those with less popular music, addressing *RQ**2* (How does the popularity of tracks within a playlist impact coherence?). This finding supports the idea that playlists containing popular music may serve more as collections of trending tracks rather than carefully curated sequences.

Contrary to our expectations, collaborative playlists, for which multiple users contribute to playlist creation, showed significant differences in coherence for only one audio feature and the artist feature. This observation touches on *RQ**4* (Do collaborative playlists, curated by more than one user, differ in terms of coherence?). These small differences might be due to the collaborators being overly passive at the time the dataset was collected – a hypothesis that needs further investigation.

Future research should explore the creation date of playlists and the time intervals between edits, as the evolving music taste of the curator is likely a significant influencing factor that could link these findings. Additionally, incorporating the curators’ personality traits and listening contexts, e.g., playlists created for specific activities, such as running, or for social settings like parties, could provide deeper insights into the factors that influence playlist curation and coherence. Further studies should also analyze collaborative playlists in more recent datasets, potentially with a different subset of coherence features, to determine whether the coherence behaviors of personal and collaborative playlists remain consistent.

In summary, our study is among the first to analyze the contributing factors for coherent playlists, providing valuable insights for improving music recommendation systems and enhancing user experiences in music streaming services.

## Supplementary Information

Below is the link to the electronic supplementary material.

(PDF 154 kB)

## Data Availability

The Million Playlist Dataset [38] analyzed during the current study are available on AICrowed [66]. Audio features and the list of artists for tracks can be retrieved using the Spotify API [42, 55]. The coherence scores of each playlist analyzed during the current study, along with the respective code, are available in the GitHub repository: https://github.com/hcai-mms/coh_analysis.

## Footnotes

1 Dubstep, an electronic dance music genre that emerged in the 2000s, is typically characterized by heavy bass lines and syncopated rhythms, with a tempo of around 140 beats per minute.

2 In the figures, the number of edits is abbreviated as #edits.

3 Alternatively, Spotify’s API can provide popularity scores for tracks. However, these scores are continuously updated to reflect the latest trends, making them less suitable for estimating the popularity of tracks in a dataset created between 2010 and 2017.

4 We deliberately chose the term personal playlist over private playlist to emphasize that, while these playlists are curated by individuals, they are still available to the public for listening.

5 In statistics, the sample variance $s^{2}$ with a denominator of $n-1$, known as the Bessel’s correction, is sometimes preferred over the population variance. For the coherence formula, the decision to use Bessel’s correction is not relevant, as this part of the denominator is canceled out in the final coherence formula.

6 Using $2 (n-1)$ instead of 2*n* would intuitively make more sense as it accounts for the number of intervals between the samples. This approach is valid if the sample variance is used instead of the population variance for the final coherence formula. Regardless of the selected version, both lead to the same coherence formula.

7 The playlists have been categorized into genres using the title given by the user.

8 The correlations are calculated directly over the attribute values, i.e., no coherence values are involved, allowing for the application of Kendall’s Tau and point-biserial correlation.

9 https://www.deezer.com.

10 Music taste defined as the “person’s slowly evolving long-term commitment to a particular music idiom” [62] is discussed in more detail, along with influencing factors, by [7].

11 The value of *α* is chosen based on the dataset size, ensuring that subsequent tracks with approximately 100 or more co-occurrences and strong evidence are considered related.

12 Alternatively, $\Delta _{trans}^{*}$ could be initialized to zero instead of −∞, ensuring that swaps are only made if they improve both coherence and transitions.

13 Original “study music”: https://open.spotify.com/playlist/7Fsw9PLi7Lw75j334ufHvF.

14 Reordered “study music”: https://open.spotify.com/playlist/5JIwSA76To12j9IqXEsbpY.

## Abbreviations

APC: Automatic Playlist Continuation
APG: Automatic Playlist Generation
ATE: Average Treatment Effect
BPM: Beats per Minute
CP: Collaborative Playlist
MIR: Music Information Retrieval
MPD: Million Playlist Dataset
MRS: Music Recommender System
PP: Personal Playlist;
