# Supplementary material for: The impact of playlist characteristics on coherence in user-curated music playlists
Source: EPJ Data Sci. 2025 Mar 19;14(1):24. doi: 10.1140/epjds/s13688-025-00531-3 (PMC11923031; doi:10.1140/epjds/s13688-025-00531-3)
Supplement: Supplementary file 1 — (PDF 154 kB) [file 13688_2025_531_MOESM1_ESM.pdf]

Supplementary information

Supplementary information accompanies this paper, including Lemma 1 and Lemma 2, which provide two examples of item sequences that minimize and maximize coherence, along with their respective proofs. Table S1 presents the results of repeated coherence experiments on shuffled data, further validating the robustness of our findings.

Lemma 1. *If* $x_i = i$ *mod* $2, i \in \mathbb{N}$ *then* $coh(\{x_i\}_i^\infty) \to -1$

Proof.

$$d(x_i, x_{i+1})^2 = (x_i - x_{i+1})^2 = \begin{cases} 1 = (0-1)^2 & \text{for i is even} \\ 1 = (1-0)^2 & \text{for i is odd} \end{cases}$$

$$d(x_i, x_j)^2 = (x_i - x_j)^2 = \begin{cases} 0 = (0-0)^2 = & \text{for } i \text{ is even and } j \text{ is even} \\ 1 = (0-1)^2 = & \text{for } i \text{ is even and } j \text{ is odd} \\ 1 = (1-0)^2 = & \text{for } i \text{ is odd and } j \text{ is even} \\ 0 = (1-1)^2 = & \text{for } i \text{ is odd and } j \text{ is odd} \end{cases}$$

$$\vec{s}^2(x_n) = \frac{1}{2n} \sum_{i=1}^{n-1} d(x_i, x_{i+1})^2 = \frac{1}{2n} \sum_{i=1}^{n-1} 1 = \frac{n-1}{2n}$$

$$\sigma^2 = \frac{1}{n^2} \sum_{i=1}^{n} \sum_{j=i+1}^{n} d(x_i, x_j)^2 = \frac{1}{2n^2} \sum_{i=1}^{n} \sum_{j=1}^{n} d(x_i, x_j)^2 = \frac{1}{2n^2} \sum_{i=1}^{n} \frac{n}{2} = \frac{1}{2n^2} \frac{n^2}{2} = \frac{1}{4}$$

$$coh = 1 - \frac{\vec{s}^2}{\sigma^2} = 1 - \frac{\frac{n-1}{2n}}{\frac{1}{4}} = 1 - \frac{2(n-1)}{n}$$

$$coh_{n\to\infty} = \lim_{n\to\infty} 1 - \frac{2(n-1)}{n} = 1 - 2 = -1$$

□

Lemma 2. *If* $x_i = \frac{i}{n}, i = 1, 2, \ldots n$ *and* $n \in \mathbb{N}$ *then* $coh(\{x_i\}_i^{\infty}) \to 1$

Proof.

$$d(x_i, x_{i+1})^2 = (x_i - x_{i+1})^2 = \left(\frac{i}{n} - \frac{i+1}{n}\right)^2 = \left(\frac{i-(i+1)}{n}\right)^2 = \frac{1}{n}^2 = \frac{1}{n^2}$$

$$d(x_i, x_j)^2 = (x_i - x_j)^2 = \left(\frac{i}{n} - \frac{j}{n}\right)^2 = \left(\frac{i-j}{n}\right)^2 = \frac{(i-j)^2}{n^2}$$

$$\sum_{i=1}^{n}\sum_{j=1}^{n} d(x_i, x_j)^2 = \sum_{i=1}^{n}\sum_{j=1}^{n} \frac{(i-j)^2}{n^2} = \frac{1}{n^2}\sum_{i=1}^{n}\sum_{j=1}^{n}(i-j)^2 = \frac{1}{n^2}\frac{n^2(n^2-1)}{6} = \frac{n^2-1}{6}$$

$$\overrightarrow{s}^2 = \frac{1}{2n}\sum_{i=1}^{n-1} d(x_i, x_{i+1})^2 = \frac{1}{2n}\sum_{i=1}^{n-1}\frac{1}{n^2} = \frac{n-1}{2n}\frac{1}{n^2} = \frac{n-1}{2n^3}$$

$$\sigma^2 = \frac{1}{n^2}\sum_{i=1}^{n}\sum_{j=i+1}^{n} d(x_i, x_j)^2 = \frac{1}{2n^2}\sum_{i=1}^{n}\sum_{j=1}^{n} d(x_i, x_j)^2 = \frac{1}{2n^2}\frac{n^2-1}{6} = \frac{n^2-1}{12n^2}$$

$$\frac{\overrightarrow{s}^2}{\sigma^2} = \frac{\frac{n-1}{2n^3}}{\frac{n^2-1}{12n^2}} = \frac{12n^2(n-1)}{2n^3(n-1)*(n+1)} = \frac{12n^2}{2n^3(n+1)} = \frac{6}{n(n+1)}$$

$$coh = 1 - \frac{\overrightarrow{s}^2}{\sigma^2} = 1 - \frac{6}{n(n+1)}$$

$$coh_{n\to\infty} = \lim_{n\to\infty} 1 - \frac{6}{n(n+1)} = 1 - 0 = 1$$

$\square$

Table S1: Results of Correlation and Causal Inference Analysis

	Length									
	Pearson			Means			ATE			
	corr	p	N	c	t_1	t_2	$c \rightarrow t_1$	$c \rightarrow t_2$	$p_{c \rightarrow t_1}$	$p_{c \rightarrow t_2}$
artists	0.000	.9805	650k	0.000	0.000	0.000	−0.001	0.001	.2339	.1590
loudness	0.000	.7439	650k	0.000	0.000	0.000	−0.003	−0.002	.1581	.3310
energy	0.000	.8023	650k	0.000	0.000	0.000	0.001	0.002	.6108	.2918
danceability	−0.001	.6191	650k	0.000	0.000	0.000	−0.001	0.000	.7613	.9907
acousticness	−0.001	.4120	650k	0.000	0.000	0.000	−0.002	−0.004	.4577	.0229
valence	0.000	.6968	650k	0.000	−0.001	0.000	0.003	−0.003	.2044	.0875
speechiness	0.000	.8937	650k	0.000	0.000	0.000	0.004	0.002	.0580	.2300
instrumentalness	0.001	.4706	583k	−0.001	0.000	0.000	−0.001	−0.002	.7468	.1992
liveness	0.000	.8420	650k	0.000	0.000	0.000	0.000	0.002	.9670	.3563
tempo	0.000	.9526	650k	0.000	0.000	0.000	−0.002	0.000	.2441	.9102
tonality	−0.001	.5915	650k	0.000	0.000	0.000	0.000	0.000	.9741	.7533

	#Edits									
	Pearson			Means			ATE			
	corr	p	N	c	t_1	t_2	$c \rightarrow t_1$	$c \rightarrow t_2$	$p_{c \rightarrow t_1}$	$p_{c \rightarrow t_2}$
artists	0.000	.8106	650k	0.000	0.000	0.000	0.000	0.002	.9146	.4316
loudness	0.000	.7775	650k	0.000	0.000	0.000	−0.001	0.001	.6645	.9021
energy	0.000	.9532	650k	−0.001	0.000	0.000	0.002	0.015	.5329	.4499
danceability	0.000	.9586	650k	0.001	0.000	0.000	0.004	0.016	.0949	.1341
acousticness	0.000	.6889	650k	0.000	0.000	0.000	−0.002	−0.021	.4574	.2057
valence	0.000	.7530	650k	0.000	0.000	0.000	−0.001	0.016	.8000	.3527
speechiness	0.000	.7911	650k	0.000	0.000	0.000	−0.004	0.007	.1508	.4377
instrumentalness	0.001	.2843	583k	−0.001	0.000	0.000	0.001	0.013	.7938	.2331
liveness	0.000	.8274	650k	0.000	0.000	0.000	−0.001	0.007	.7093	.6497
tempo	0.001	.5056	650k	0.000	0.000	0.000	0.001	0.001	.6967	.9246
tonality	−0.001	.2495	650k	0.000	0.000	0.000	−0.001	−0.013	.5909	.1184

	Track popularity									
	Pearson			Means			ATE			
	corr	p	N	c	t_1	t_2	$c \rightarrow t_1$	$c \rightarrow t_2$	$p_{c \rightarrow t_1}$	$p_{c \rightarrow t_2}$
artists	−0.001	.4698	650k	0.000	0.000	0.000	0.000	0.000	.3168	.4848
loudness	0.000	.9626	650k	0.000	0.000	0.000	0.003	0.000	.1716	.8196
energy	0.000	.9608	650k	0.000	0.000	0.000	0.002	0.000	.4160	.9645
danceability	0.000	.9017	650k	0.000	0.000	0.000	0.000	0.001	.9659	.6288
acousticness	−0.002	.1940	650k	0.001	−0.001	0.000	−0.001	0.001	.4971	.7640
valence	0.000	.8604	650k	0.000	0.000	0.000	−0.002	−0.001	.3115	.5178
speechiness	−0.001	.5055	650k	0.000	0.000	0.000	−0.001	0.000	.6551	.7788
instrumentalness	0.001	.2595	583k	−0.001	0.000	−0.001	0.002	−0.002	.2534	.1173
liveness	−0.002	.0691	650k	0.001	0.000	0.000	0.001	0.000	.4749	.9107
tempo	0.001	.6756	650k	0.000	0.000	0.000	−0.001	−0.003	.7655	.1272
tonality	−0.001	.6677	650k	0.000	0.000	0.000	0.000	−0.001	.8533	.3720

	Collaborative						
	Point-biserial			Means		ATE	
	corr	p	N	c	t_1	$c \rightarrow t_1$	$p_{c \rightarrow t_1}$
artists	−0.001	.6188	650k	0.000	0.000	0.001	.1872
loudness	0.001	.2760	650k	0.000	0.002	−0.001	.5527
energy	0.002	.1109	650k	0.000	0.002	0.003	.2559
danceability	0.001	.4723	650k	0.000	0.001	0.001	.8173
acousticness	0.000	.8332	650k	0.000	0.000	−0.003	.1497
valence	−0.001	.5188	650k	0.000	−0.001	−0.003	.1448
speechiness	0.000	.9303	650k	0.000	0.000	0.000	.8167
instrumentalness	−0.001	.2595	583k	0.000	−0.002	−0.003	.0769
liveness	0.001	.6376	650k	0.000	0.001	−0.003	.2722
tempo	−0.001	.5790	650k	0.000	−0.001	−0.003	.1725
tonality	−0.001	.6081	650k	0.000	0.000	0.000	.9543

The content and experimental setup mirror those of Table 4 in the article, but with tracks shuffled within each playlist. This experiment serves as a validity check, ensuring that coherence results from both the correlation and causal inference experiments become insignificant and that the means converge to zero as expected, when the playlist track order is randomized.
